# A Unified Model of Shoot Tropism in Plants: Photo-, Gravi- and Propio-ception

**DOI:** 10.1371/journal.pcbi.1004037

**Published:** 2015-02-18

**Authors:** Renaud Bastien, Stéphane Douady, Bruno Moulia

**Affiliations:** 1 Institut Jean-Pierre Bourgin, UMR1318 INRA-AgroParisTech, 78026 Versailles, France; 2 INRA, UMR 547 PIAF, F-63100 Clermont Fd Cedex 01, France; 3 Clermont Université, Université Blaise Pascal, UMR 547 PIAF, BP 10448, F-63000 Clermont-Ferrand, France; 4 School of Engineering and Applied Sciences, Harvard University, Cambridge, MA 02138, USA; 5 Matière et Systèmes Complexes, Université Paris-Diderot, 75025 Paris Cedex 13, France; University of Notre Dame, UNITED STATES

## Abstract

Land plants rely mainly on gravitropism and phototropism to control their posture and spatial orientation. In natural conditions, these two major tropisms act concurrently to create a photogravitropic equilibrium in the responsive organ. Recently, a parsimonious model was developed that accurately predicted the complete gravitropic and proprioceptive control over the movement of different organs in different species in response to gravitational stimuli. Here we show that the framework of this unifying graviproprioceptive model can be readily extended to include phototropism. The interaction between gravitropism and phototropism results in an alignment of the apical part of the organ toward a photogravitropic set-point angle. This angle is determined by a combination of the two directional stimuli, gravity and light, weighted by the ratio between the gravi- and photo-sensitivities of the plant organ. In the model, two dimensionless numbers, the graviproprioceptive number B and the photograviceptive number M, control the dynamics and the shapes of the movement. The extended model agrees well with two sets of detailed quantitative data on photogravitropic equilibrium in oat coleoptiles. It is demonstrated that the influence of light intensity I can be included in the model in a power-law-dependent relationship M(I). The numbers B and M and the related photograviceptive number D are all quantitative genetic traits that can be measured in a straightforward manner, opening the way to the phenotyping of molecular and mechanical aspects of shoot tropism.

## Author Summary

Although plants are mostly seen as static, they are constantly moving to adapt to changes in their stature and to their environment. Gravity and light, among others, are major factors that sculpt the shapes of plants. Plants tend to grow in the direction of the light to get access to their energy resource. At the same time, however, they need to maintain their balance and control their posture. In a recent study, we showed that postural control is regulated by two types of perception: graviception and proprioception. We extend that study to include light perception in order to obtain a unified description of plant tropism. As the system is highly dynamic, a model is required in order to evaluate hypotheses against experimental data. Our results show that the direction of plant growth is determined by the combined influence of light and gravity, whereas postural control is still regulated by gravity and proprioception.

## Introduction

Plants are constantly moving to reach the light and to maintain the architecture and posture of their aerial organs: stems, branches, leaves… These movements are active, generally powered by differential growth and are controlled by environmental signals [1]. Tropisms are the class of movements that are oriented by a vectorial environmental factor. Light is the main source of energy for plants and is a major cue for tropic movement. In phototropism, shoots grow in the direction of the light source. Under natural conditions on Earth, gravity is unavoidable and drives gravitropism, in which shoots usually grow against the direction of gravity. Proprioception, the ability of plants to perceive their own deformations, has recently been identified as a major factor in tropic movement; it stimulates the straightening of a curved organ, a response that can be thought of as autotropism [2–4]. Such classification of control mechanisms into three different tropic drivers is however merely a conceptual convention: it is likely that, in natural conditions, the three processes interact constantly. Plant shoots generally exhibit negative gravitropism, positive phototropism, and negative autotropism in the same organs at the same time.

The way phototropism and gravitropism interact to control the movement of plant shoots is acknowledged to be important in determining plant habit in natural conditions but is not well understood [5]. Advanced genetic studies in the model species *Arabidopsis thaliana*, which used mutants with impaired gravitropism or phototropism [6, 7], have shown that the two processes interact in a complex manner to control movement in wild-type plants [8]. However, part of this complexity lies in the variety of phototropic responses observed in response to different qualities and intensities of the light stimulus. For example, the effects of low-fluence pulses differ from those of continuous light [7]; but only the latter are directly relevant to plant growth under natural conditions, and will therefore be considered here.

The focus of the current study is the regulation and the control of organ movements during the interaction of tropisms. To do so, it is proposed to extend the recent dynamical model for gravitropism called the *AC* model [2] to phototropism. In this model, active tropic bending is controlled by the additive (but opposing) effects of graviception and proprioception, expressed by

$$\frac{\partial C(s,t)}{\partial t}=-\beta A\left(s,t\right)-\gamma C\left(s,t\right)\;\text{for}\;s>L-L_{gz}\;\text{and}\;0\;\text{otherwise}$$ (1)

where *s* is the position along the organ, *L*_*gz*_ is the length of the growth zone, *L* is the length of the entire organ, *t* is time, *A*(*s*, *t*) is the local angle of the organ to the vertical, *C*(*s*, *t*) is the local curvature (i.e., the spatial rate of change of *A* along *s*) and the parameters *β* and *γ* are, respectively, the gravi- and proprio-ceptive sensitivities. The *AC* model was shown to explain the complex kinematics of gravitropism in eleven species covering a broad taxonomical range of angiosperms, major growth habits and organ types. [3, 4]

Although graviception and proprioception act additively, their control over the dynamics of tropic movement and the steady-state final shape actually depends only on the ratio between gravisentitivity and propriosentitivity, scaled to the size of the growth zone. In the model, this is formalized through the definition of the dimensionless graviproprioceptive bending number *B* = *β L*_*gz*_/*γ. B* fully defines both the time to reach the steady state and the final shape at steady state, and can be measured in simple morphometric experiments. As *B* is dimensionless, it can be used to make quantitative comparisons between experiments involving very different sizes and growth velocities. This enables universal behaviors and control mechanisms to be identified.

To understand and describe properly the interaction between gravitropism and phototropism, successive steps are undertaken: First, the hypotheses that lead the construction of the model are discussed, then general specifications of the geometry of the organ and of the gravity and light fields are established. In the following part, the construction of model is given. Starting with the simple case of phototropism in isolation which allows to dissect the interaction between photosensitivity and propriosensitivity. Finally, the dynamics of the interaction among photoception, graviception and proprioception are explored, including the influence of light intensity. All these models are investigated for different localization of photoception, at the tip or distributed along the organ. Quantitative experimental data on photogravitropic equilibrium are used to test the validity of the model. These results and the implications for plant biology are discussed.

## Materials and Methods

Before presenting the construction of a dynamic model, it is important to state properly what are the hypotheses that lead our work, as well as the simplified geometries of the organ and of the source fields that are considered. Once these bases are well defined, we will discuss the construction of successive model variants of increasing complexity, first with the simple phototropic model, then with the model describing the interaction between gravitropism and phototropism. For each model two sub-models are considered, depending on the distribution of the photoception along the organ.

### Hypotheses

In order to formulate hypotheses regarding the extension of the AC model to include phototropism, it is now useful to review our knowledge of the distribution of sensing mechanisms and differential growth responses along the plant organ, the mechanisms of photo- gravi- and proprio-ception, as well as their possible interactions.

The localization of gravisensitivity has been established in more detail than the localization of photosensitivity. The plant perception of gravity is related to the presence of statoliths within specialized cells called statocysts. In aerial organs, statocysts and statoliths are found throughout the growth zone, and both perception and the bending response are local [9]. Our knowledge of the mechanisms of proprioception is limited, but it is known to be exclusively local and likely to involve cytoskeleton remodeling [1, 2].

In phototropism, blue-light-sensitive phototropins sense the direction of incoming light, but they interact with other photoreceptors in a network that remains to be fully elucidated [10–13]. Photoreceptor localization studies were pioneered by Darwin [14]. By masking different parts of coleoptiles, Darwin found that the perception of light occurred at the coleoptile apex. As tropic bending occurred all along the growth zone of the organ, a secondary basipetal signal was postulated to be involved, now elucidated as a lateral redistribution of the polar transport of the plant hormone auxin [10]. Very few studies have further systematically analyzed the localization of photoreceptors or even phototropic responses along plant organs. It has been found, however, that apical phototropic sensing is not universal. For example, the hypocotyl of *Arabidopsis* exhibits photoreceptors all along the growth zone, allowing for distributed local photoception and local phototropic growth responses [11, 15]. The functional significance of such differences in the localization of photosensitivity among organs has not been investigated.

A few quantitative studies of the effects of continuous light [16–18] have shown that phototropism and gravitropism seem to act in an additive manner. When a plant organ is lit from a direction that differs from that of gravity, the plant undergoes an active movement until a steady-state shape called the photogravitropic equilibrium is reached [17, 19]. The photogravitropic equilibrium angle (*PGEA*) of the organ tip was found to follow the phenomenological equation

$$PGEA=k\;\log\left(\frac{I}{I_{0}}\right)-g\;\sin A_{0}$$ (2)

where *I* is the fluence rate of illumination, *I*_0_ is the light-sensing threshold, *g* is the gravitational force, and *A*_0_ is the initial angle of inclination towards gravity. The term *g*sin*A*_0_ is the sine law of gravitropism [4]. This sine law was also used when defining the gravisensing term in the *AC* model for gravitropism1 but in this case it has been reduced to a linear term using the approximation sin(*A*(*s*, *t*)) ≈ *A*(*s*, *t*) + *O*(*A* ^3^). The term *k* *log*(*I*/*I*_0_) reflects the phototropic stimuli. Both phototropic and gravitropic sensing have been shown to control the relocalization of polar auxin transporters, controlling the formation of lateral gradients in auxin concentration and hence differential growth. These molecular dynamics might explain why the effects of the two stimuli on PGEA are additive [7, 20, 21]. Thus far, the dynamics of the processes leading to this photogravitropic equilibrium have not been analyzed or modeled in detail.

Building on this current knowledge, we propose herein to extend the graviproprioceptive *AC* modeling approach to study phototropism and its interactions with gravitropism. We focus on investigating dynamic control of photosensitivity, gravisensitivity and propriosensitivity throughout the whole movement.

More precisely, our working hypotheses are as follows:

(H1) The action of the tropic motor is fully driven by the perception-regulation process and results in a change in the local curvature.

(H2) The angles formed between the axis of the growing organ and the respective gravity and light fields influence graviception and photoception, and hence influence gravity- and light components driving the tropic response.

(H3) Proprioception takes place regardless of whether external tropic signals are present. The occurrence of proprioception has been shown for gravitropism [2, 22]. Concerning phototropism, plants growing in a clinostat (an apparatus that suppresses graviception) were also shown to straighten after a transient light pulse [23]. Thus, we assume that each constituent element of the organ perceives its own local deformation during active bending, namely the curvature, and responds in order to restore local straightness [2].

(H4) The different types of perception act additively within the model, meaning that their respective contributions have equivalent roles in driving the movement. It is therefore expected that much of the apparent complexity of the motion is due to spatio-temporal integration of several responses over the changing geometry of the organ with respect to the light and gravity fields.

In order to formulate testable and refutable predictions regarding the dynamic control of tropic movement, we combine these working hypotheses into successive variants of a dynamic model, extending the graviproprioceptive *AC* modeling approach. These models are expected to provide relevant dimensionless numbers that control the dynamics, as well as a means of estimating their values on the basis of experimental measurements. This will enable the model and hypotheses to be evaluated experimentally, and eventually open the way to obtaining accurate phenotypes of tropisms.

We have chosen to neglect the effects related to the elongation of the organ. Recent work has shown that elongation tends to destabilize tropic movements and should increase the oscillations that take place during these movements [24]. We have found, however, that the proprioceptive sensitivities of modern angiosperms have evolved such that these effects are fully controlled by the plants. Therefore, these growth-related effects can be neglected in the description of tropic movement [24]. Similarly, even though plants are expected to bend under their own weight, we propose that the effects of a plant’s weight on its shape can also be neglected [25]. This is based on the fact that the *AC* model, which takes into account the plant’s perception as a sole driving process, has proved to be sufficient to explain the movement of a wide variety of organs and species of many orders of magnitude of size.

To finish, it is important to note that the *AC* model accounts for orthogravitropism, wherein the organ aligns with the direction of gravity. Some organs align in a direction different from that of gravity, called the gravitropic set-point angle (GSA) [26, 27]. It is possible to adapt the *AC* model for such cases by modifying the model’s graviceptive term, −*β A*(*s*, *t*)→−*β* (*A*(*s*, *t*)−*GSA*) such that the organ aligns with the GSA. However, the *AC* model has not been tested for non-orthogravitropic organs. And this study concentrates on the case of ortho-gravi and photo-tropisms (a very common case in primary shoots).

As stated before, the present study involve successive variants of the model. To unease their handling we named the successive models presented below according to i) the sensory processes that are involved and their locations (biological specification)—e.g., photoproprioceptive; and ii) the driving variables under investigation (mathematical specification) (in accordance with the naming convention in [2], *e.g.* model AC is driven par the inclination angle A versus the vertical and the curvature C all along the axis, whereas model A^a^C is driven by the apical angle of inclination and by the curvature all along the axis). The reader may find a list of name of the variables and parameters in Table 1 and of the model in Table 2.

**Table 1:** Variables and Parameters.

*s*	Curvilinear abscissa from the base to the apex
*t*	Time
*L*	Length of the organ
*L*_*c*_	Length of the curved zone
*A*(*s*, *t*)	Local angle
*C*(*s*, *t*)	Local curvature
*β*	Graviceptive sensitivity
*γ*	Proprioceptive sensitivity
*ν*	Photoceptive sensitivity
$B=\frac{\beta L}{\gamma }$	Graviproprioceptive number
$D=\frac{\nu L}{\gamma }$	Photoproprioceptive number
$M=\frac{\nu }{\beta }$	Photograviceptive number
*A*_0_	Initial angle of the organ
*A*_*P*_	Orientation angle of the light
$A_{R}=\frac{A_{P}}{1+M}$	Resultant photogravitropic orientation angle
*A* ^*a*^ = *A*(*L*, *t*)	apical angle of the organ
$A_{R}^{a}=A(L,t)-A_{R}$	Resultant photogravitropic orientation apical angle
*I*	Intensity of the light
Φ_*S*_(*I*) = *aI* ^*b*^	relation between the intensity of the light and the observed response. Stevens law.
Φ_*F*_(*I*) = *c* + *d* log(*I*)	relation between the intensity of the light and the observed response. Weber-Fechner law.
*GSA*	Gravitropic Setpoint Angle
*PGSA* = *A*_*R*_	PhotoGravitropic Setpoint Angle
*PGEA*	PhotoGravitropic Equilibrium Angle

**Table 2:** Models.

*name*	*notation*	*equation*
graviproprioceptive	*AC*	$\frac{\partial C(s,t)}{\partial t}=-\beta A(s,t)-\gamma C(s,t)$
photoproprioceptive apical	*A* ^*a*^ *C*	$\frac{\partial C(s,t)}{\partial t}=-\nu A(L,t)-\gamma C(s,t)$
photograviproprioceptive	*A*_*R*_ *C*	$\frac{\partial C(s,t)}{\partial t}=-\beta A(s,t)-\nu (A(s,t)-A_{P})-\gamma C(s,t)$
photo-gravi-proprioceptive apical	$A_{R}^{a}C$	$\frac{\partial C(s,t)}{\partial t}=-\beta A(s,t)-\nu (A(L,t)-A_{P})-\gamma C(s,t)$

The different models used in this paper, with their full names and their respective notations.

### Geometrical specifications of the organ and of the source fields

One drawback of most studies on phototropism is that they have overlooked the true geometry of the organ, measuring only the orientation of the apical tip of the organ [28–31]. One of the greatest insight of the *AC* model was to show that the geometry of the organ and of the source field is central to describe the movement properly. It is then important to have a clear description of the studied geometry.

**Figure 1:**
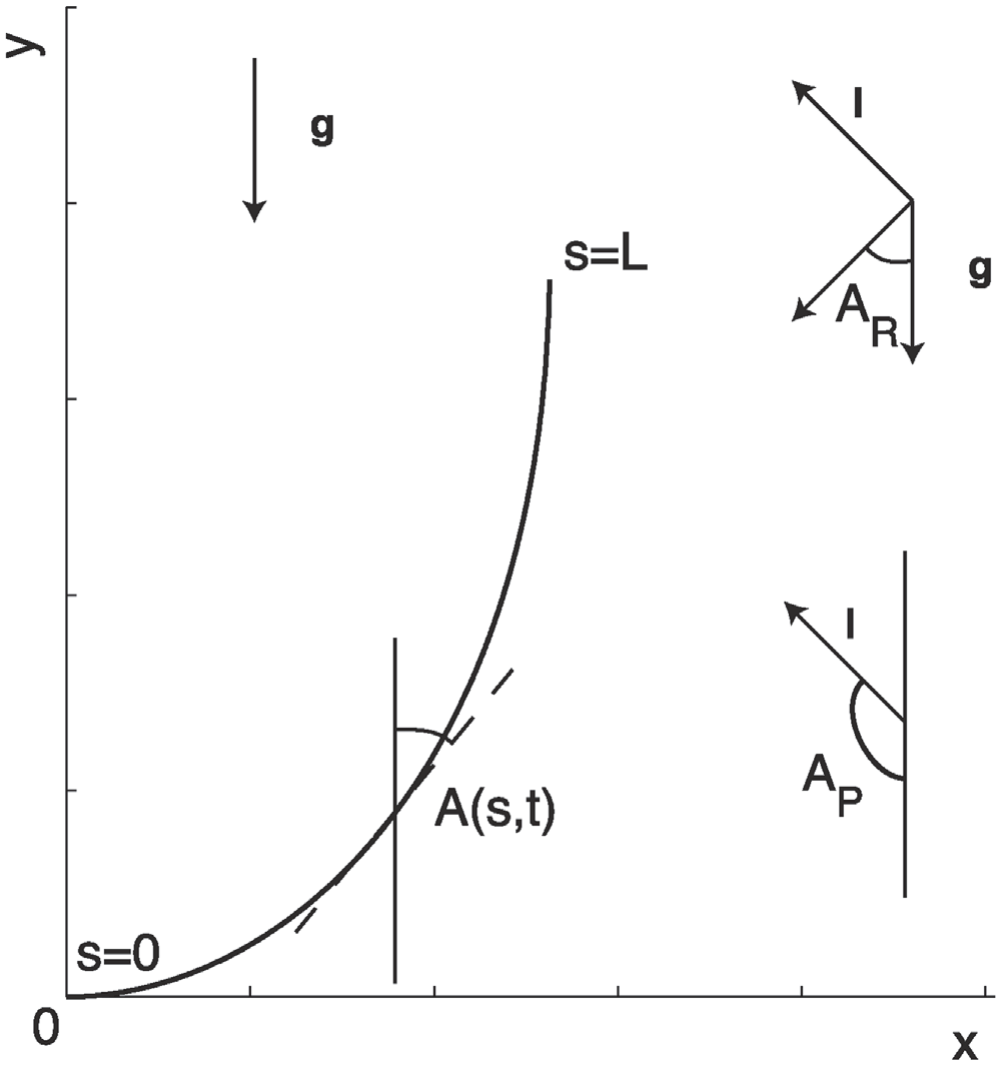
Geometric description of organ shape. The median line of an organ of total length *L* is in a plane defined by coordinates *x*, *y*. The arc length *s* is defined along the median line with *s* = 0 referring to the base and *s* = *L* referring to the apex. *A*(*s*) is the local orientation of the organ with respect to the vertical, and *C*(*s*) the local curvature. The orientation of the gravity field vector *g* is parallel to the *y* axis. The orientation of the light field vector *l* forms an angle *A*_*P*_ with the *y* axis. The resulting angle of the two tropisms is called *A*_*R*_.

Like the *AC* model, the models developed herein describe the shape of the organ in terms of its median line, i.e., its central axis (Fig. 1). We parameterize the position along this median line according to the curvilinear abscissa *s* going from the base *s* = 0 to the apex *s* = *L*. The angle *A*(*s*, *t*) then describes the local orientation of the median with respect to the vertical pointing up (and hence to the direction of the gravity vector **g**) at time *t*. The angle of the apex at the tip of the organ, *A* ^*a*^, is then equal to *A*(*L*, *t*). The local curvature *C*(*s*, *t*) is the spatial rate of change of *A*(*s*, *t*) along *s*, and from differential geometry we know that

$$C\left(s,t\right)=\frac{\partial A(s,t)}{\partial s}\;\;\mathrm{or}\;\;A\left(s,t\right)=A_{0}+\int_{0}^{s}C\left(l,t\right)dl$$ (3)

At the scale of the plant, gravity acceleration **g** is homogeneous, uniform, and invariant to translation and to rotation around the main direction of the gravity field (Fig. 1). The case of light perception seems to be less straightforward. The geometry of light fields in nature can be quite diverse. At the scale of the plant, the light field produced by the sun on the earth is supposed to be homogeneous, uniform, and invariant to translation and to rotation around the main direction of the light field, but its orientation relative to the gravity field varies over the course of the day. In the lab, a point light source can emit a spherical field whose intensity (irradiance) decreases with the square of the distance. Furthermore, multiple point sources might be used, thereby increasing the complexity of the analysis. Thus far, most controlled experiments have used a distant punctual source with a collimated beam [16–18, 32]. For simplicity, we assume that the light field **l** has the same symmetry as the gravity field. This assumption holds true if the source of light is far from the organ. To further reduce the complexity of the analysis, we do not consider curvature outside the plane; that is, we explore the case in which the main direction of the plant’s organ, the gravity field **g** and the light field **l** are all in the same plane *P*_*gl*_ (Fig. 1). This again was the case in most controlled experimental studies (e.g. [17]). Therefore, the direction of the lighting in the *P*_*gl*_ plane is defined by the angle *A*_*p*_ and by a radiance (or light intensity) *I*_0_ (Fig. 1).

The simplifications we adopt enable us to assess the model by comparing it with experimental data, while still providing insights regarding the regulation of the movement. Moreover, the assumption that the initial direction of the organ, the direction of gravity and that of the light are in the same plane is fulfilled for organs that are already aligned with the direction of gravity.

### Optical aspects of photoception

Coleoptiles and other plant shoots appear to sense light direction via the light gradient across the organ [33]. Treatments that change the steepness of this gradient (e.g., infiltration with dyes or changes in the amounts of natural pigments) alter the phototropic response: the steeper the gradient, the more the coleoptile bends towards the light. This gradient depends on internal light transfer through plant tissues but also depends linearly on the irradiance of the light impinging on one side of the surface of an organ. This irradiance is given by Lambert’s cosine law of irradiance of geometrical optics (e.g. [17]), which states that the irradiance falling on any surface varies in proportion to the cosine of the incident angle.

$$I\left(s,A_{P}\right)=I_{0}\;\cos\left(\frac{\pi }{2}-\left(A\left(s,t\right)-A_{P}\right)\right)=-I_{0}\;\sin\left(A\left(s,t\right)-A_{P}\right)$$ (4)

As the *AC* model is a first-order model, and in the limit of small angles *A*(*s*, *t*)−*A*_*P*_, we can use the approximation sin(*A*(*s*, *t*)−*A*_*P*_) ≈ *A*(*s*, *t*)−*A*_*P*_ + *O*(*A* ^3^), so that the photosensitivity is proportional to the angle between the organ and the light direction i.e., *A*(*s*, *t*)−*A*_*P*_ (the influence of the intensity of the light irradiance will be considered in a later section). Although this approximation is only valid for small angles, such an approximation gives a good and efficient understanding of the dynamics of the system. The influence of each term on the tropic movement can be easily understood and discussed.

### Phototropism

We may now start with the simplest case of phototropism without gravitropism. Using equation 4, a phototropic model can be constructed. Two sub-models are considered, when the perception is apical and when the perception is local.

#### Apical photoception: *A^a^C* model

In cases where perception of light is found to be purely apical, the previous dependency, equation 4, is expressed as *A*(*L*, *t*)−*A*_*P*_. The photosensitivity corresponding to the inclination of the apex in relation to the light beam is translated into a secondary signal, propagated basipetally along the organ through asymmetric polar transport of the plant hormone auxin [10]. As auxin transport is faster than the characteristic time of the movement, this long-distance signaling along the organ can be approximated as instantaneous, so that, at any position *s*, a small segment of the organ is submitted to a signal proportional to *A*(*L*, *t*)−*A*_*P*_ (SI). Indeed, it can be shown that the propagation of the signal has only slight effects on the dynamics and the steady state of the organ, as long as the characteristic time of propagation (the time for the signal to go from the apex to the base) is smaller than the characteristic time of movement (given by the elongation rate [25]) (SI). The perceived signal is thus the value at the apex, *A*(*L*, *t*), and this is the only value accessible along the organ.

Let us now assume for a moment that the light is zenithal, *A*_*P*_ = 0, so that the phototropic reaction tends to bring the tip to the vertical.

The conditions of symmetry described in [2] are then fulfilled. The behavior remains the same, according to the change *A*(*s*, *t*) → −*A*(*s*, *t*) and then *C*(*s*, *t*) → −*C*(*s*, *t*).

According to hypotheses H1–4, in the case of zero gravity or zero graviception (agravitropic mutants), the most basic linear equation for an apical perception can then be formulated as

$$\frac{\partial C(s,t)}{\partial t}=-\nu A\left(L,t\right)-\gamma C\left(s,t\right)$$ (5)

where *νA*(*L*, *t*) is the apical photoceptive term, readily transmitted all along the organ, and *γ C*(*s*, *t*) is the proprioceptive term. This will be referred to as the apical photo-proprioceptive model, or the *A^a^C* model, with ^*a*^ expressing the apical perception of the stimulus (see Table 1). As noted, this model assumes that the transmission is instantaneous. Every part of the organ is able at any point to perceive the exact orientation of the apex. It is also assumed that the transmission is homogenous along the coleoptile, meaning that *ν* is not dependent on the position *s* (see SI).

Now let us assume the following initial conditions: a straight but tilted organ and the boundary conditions of perfect basal clamping:

$$A\left(s,0\right)=A\left(0,t\right)=A_{0}\;\;\;\;\;\;\;\;C\left(0,t\right)=0$$ (6)

It is easy to see that the *A^a^C* model is in fact independent of space coordinate *s*. First, the organ is initially straight, so the initial curvature is the same everywhere. Then as the perception of the light is purely apical, at any given time all parts of the organ receive the same signal, proportional to the apical orientation at that time. The variation of curvature, and hence the curvature, are the same at any point of the organ. The solution of the *A^a^* model is then given by

$$A(s,t)=A(t)\;=\;A_{0}\left(1-\frac{s}{L}\frac{1}{1+\frac{\gamma }{\nu L}}\left(1-e^{-\left(\nu L+\gamma \right)t}\right)\right)$$ (7)

$$C(s,\;t)=C(t)\;=\;-\frac{A_{0}}{L}\frac{1}{1+\frac{\gamma }{\nu L}}\left(1-e^{-\left(\nu L+\gamma \right)t}\right)$$ (8)

It is possible to define a dimensionless number *D*, the photoproprioceptive number, that expresses the ratio between photoception and proprioception:

$$D=\frac{\nu L}{\gamma }$$ (9)

#### Local photoception: *AC* model

If the perception of light is set to be local (as observed, for example, in *Arabidopsis* hypocotyls), equation 5 should be modified to the local photoceptive equation

$$\frac{\partial C(s,t)}{\partial t}=-\nu A\left(s,t\right)-\gamma C\left(s,t\right)$$ (10)

with the same initial conditions as described in equation 6. Equation 11, which describes the photoceptive model, is now strictly equivalent to the graviproprioceptive equation 1, i.e., the *AC* model [2].

### Photo-gravitropic interaction

The previous model can now be easily extended to take into account the perceptions of both light and gravity. As described in Fig. 1.A, the direction of the light stimulus is now considered to form an angle *A*_*P*_ with the vertical, the direction of the gravity. The orientation angle *A*(*s*, *t*) along the organ is still measured with respect to the vertical, and hence to the direction of gravity.

If *A*_*P*_ ≠ 0 the symmetry of the system is broken. When only one of the two tropisms influences the system, rotation around the axis defined by the direction of the corresponding field should have no effect on the system. When the two tropisms act simultaneously, however, the angle between the two fields prevents such global symmetry. It should also be noted that if the gravitropic term or the phototropic term tends to 0, the system converges to the photo-proprioceptive models outlined above.

As for the phototropic models, effects due to local and apical perception are considered and discussed.

#### Local perception: *A_R_C* model

When the organ lies in the plane defined by the direction of gravity and of light, the local equation of purely local photogravitropism, called the local photo-gravi-proprioceptive equation, is given by

$$\frac{\partial C(s,t)}{\partial t}=-\nu \left(A\left(s,t\right)-A_{P}\right)-\beta A\left(s,t\right)-\gamma C\left(s,t\right)$$ (11)

The two external tropisms act in the same way through perception of the local angle, except for the constant angle *A*_*P*_, indicating the direction of phototropism.

Simple variable substitution can be performed to simplify equation 12:

$$A^{\prime }\left(s,t\right)=A\left(s,t\right)-A_{P}\frac{\nu }{\nu +\beta }=A\left(s,t\right)-A_{R}\;\;\;\;\;C^{\prime }\left(s,t\right)=\frac{dA^{\prime }\left(s,t\right)}{dt}=C\left(s,t\right)$$ (12)

where *A*_*R*_ is a Resultant Angle linked to the balance of the two tropic factors through combined gravi- and photo-ception

$$A_{R}=A_{P}\frac{\nu }{\nu +\beta }$$ (13)

Equation 12 can be rewritten in a more compact form

$$\frac{\partial C(s,t)}{\partial t}=-\left(\nu +\beta \right)A^{\prime }\left(s,t\right)-\gamma C\left(s,t\right)$$ (14)

with no constant term. Starting with the set of initial conditions defined in equation 6, the initial conditions to be considered are then

$$A^{\prime }\left(s,0\right)=A^{\prime }\left(s,t\right)=A_{0}^{\prime }=A_{0}-A_{R}\;\;\;\;\;\;\;\;C^{\prime }\left(0,t\right)=C\left(0,t\right)=0$$ (15)

The variable substitutions and calculations shown above are equivalent to the case in which the plant has been rotated at an angle *A*_*R*_, such that the two tropisms then act together in the same direction. The local photo-gravi-proprioceptive equation 15 can thus be named the *A_R_C* model.

Another dimensionless number *M*, called the photograviceptive number, can then be defined as the ratio between the gravisensitivity and the photosensitivity:

$$M=\frac{\beta }{\nu }$$ (16)

Note that *M* is not independent from the graviproprioceptive number *B* and the photoproprioceptive number *D* but instead can be expressed as

$$M=\frac{B}{D}$$ (17)

#### Apical photoception: $A_{R}^{a}C$ model

In the case in which the perception of light is apical, the equation that describes this apical-photo/local-gravi-proprioception-driven movement, called the $A_{R}^{a}C$ model, is given by

$$\frac{\partial C(s,t)}{\partial t}=-\nu \left(A\left(L,t\right)-A_{P}\right)-\beta A\left(s,t\right)-\gamma C\left(s,t\right)$$ (18)

Using the variable substitutions shown in equation 13, the dynamical equation 19 can be simplified to

$$\frac{\partial C(s,t)}{\partial t}=-\nu A^{\prime }\left(L,t\right)-\beta A^{\prime }\left(s,t\right)-\gamma C\left(s,t\right)$$ (19)

This equation converges to a steady-state shape that is described as

$$A^{\prime }\left(s,t\to \infty \right)=A_{0}^{\prime }\left(e^{-Bs/L}-e^{-B}\frac{1-e^{-Bs/L}}{M+\left(1-e^{-B}\right)}\right)$$ (20)

$$C\left(s,t\to \infty \right)=-A_{0}^{\prime }\frac{B}{L}e^{-Bs/L}\left(1+\frac{e^{-B}}{M+\left(1-e^{-B}\right)}\right)$$ (21)

#### Apical photoception and light intensity in the *A_R_C* model

The sensitivity of the outputs of the photo-gravi-proprioceptive models *A_R_C* and $A_{R}^{a}C$ can vary as a function of the photoceptive, graviceptive or proprioceptive sensitivities (captured in the dimensionless ratios *D*, *B*, and *M*). Variations in these sensitivities could be due, for example, to natural or artificial genetic variations within or across species, such as those reported by [2] for *B*. However, the photograviceptive number, *M*, may also depend on a current environmental factor, namely, the intensity of the light *I* (in addition to the angle with the incident light direction *A*_*p*_). Many studies have indeed reported that the light fluence rate (irradiance) *I* influences the rate and strength of the phototropic reaction (e.g [17, 34]). It is thus expected that the photoceptive term *ν* in our model, and therefore the photograviceptive number *M*, should be a function of *I*, the fluence rate of the incident light beam. For example, [34] proposed a biochemical model of photoception in which the rate constant of the primary photosensitive reaction is proportional to the fluence rate *I* reaching the photosensitive tissues. The situation may be even more complex, as there have been reports of a “tonic” effect of light intensity on gravisensitivity (acting through other photoreceptors besides phototropins) [8]. Therefore, in cases in which the intensity of the incident light is subject to change, it seems that at least the photograviceptive number *M* should depend on the intensity of the incident light. Clearly, however, this dependence has yet to be quantitatively and experimentally elucidated. Several forms for the intensity dependency has been proposed in the literature but there are no clear theoretical or empirical reasons for choosing one specific formulation over another. The photobiology literature has classically assumed that this relation is given by the linear relation between the stimulus and the plant’s response, the Bunsen-Roscoe reciprocity law [35–37]. However, this law seems to be valid for only a small range of light dosage levels. The Schwarzschild law (a power-law generalization of the reciprocity law) appears to more accurately describe the relationship between the response and the intensity of the light stimuli, for a wide range of materials and systems [38] and has been found to be a fairly generic approximation, where the Bunsen-Roscoe reciprocity law can be seen as a special case of the power law. Galland [17] used a logarithm relation to express the relation between the observed *PGEA* and the intensity of the light, but [17] does not fully assess or discuss whether the assumption of a logarithmic relation is indeed preferable to the use of a power law.

The lack of an unequivocal formulation of the dependency on the intensity of the signal is also reflected in the broader domain of sensory biology, where two competing general models of the relation between sensation and stimuli from sensory biology are considered. The first is the Weber-Fechner Law [39], which postulates that the intensity of the response is proportional to the logarithm of the stimulus. The second is Stevens’ law, which postulates a power law relationship between the stimulus and the sensation [40]. Although these relations have been assessed primarily in the domain of human behavior, quantitative similarities in sensing relations between animals and plants have been identified [22]. The question of which model better describes the effect of the intensity of the stimulus (in our case light) thus remains open, and the adoption of a given law seems to be mostly dependent on the context and on the conventions of the discipline. Therefore, in what follows, we will consider the two types of formulations (log or power law) in the following and will subsequently compare them using experimental data.

We may now extend the photograviproprioceptive model by incorporating a new working hypothesis stating that

(H5) the photograviceptive number *M* is a function of the fluence rate of the incident light, i.e.,

M∼Φ(I) (22)

This yields light-intensity-dependent photo-gravi-proprioceptive models *A_R_*(*I*)*C* and $A_{R}^{a}(I)C$. The function Φ can be expressed either by a power law

$$\Phi_{S}\left(I\right)=aI^{b}$$ (23)

or by logarithmic relation

$$\Phi_{F}\left(I\right)=c+d\;\log\left(I\right)$$ (24)

### Experiments

A series of experiments were conducted or reprocessed from the bibliography in order to assess the Photo-Gravitropic models against experimental data in continuous light and 1g conditions (i.e.reminiscent of natural outdoor conditions on Earth): an experiment in the typical set-up used for phototropic studies, but tracking the entire kinematics of the movement (LLE experiment) and a set of experiments conducted by [17] and focusing only on the PhotoGravitropic Equilibrium tip Angle (PGEA), but at various tilting angles and light intensities (referred to as “Galland’s experiments” in the following).

#### Lateral light experiments with Wheat coleoptile (LLE experiment)

In order to asses the effect of lateral light on vertical organs, experiments were conducted on 12 etiolated wheat coleoptiles (Recital). Wheat seeds were grown in in vermiculite in the dark at 23°*C* until their size reached at least 2*cm* (approximatively 72h). In order to avoid effects of already curved coleoptiles, the straightest coleoptiles were selected for the experiment. The coleoptiles were then displayed in front of a camera (Nikon DS5200) with a source of blue light (LED VAOL-5701SBY4-ND collimated with a parabolic mirror RCphotonics) perpendicular to the vertical. Timelapse were taken every five minutes for 24h.

#### Galland experiments

Galland carried out a detailed quantitative study of the relationship between the *PGEA* (equation 2) and the fluence rate *I* in light-grown *Avena* coleoptiles under continuous light [17], hence contrary to our experiment the detailed kinematics were not reported. In these experiments, the coleoptiles were tilted at different angles from the vertical while the tip of each coleoptile was illuminated orthogonally and in the plane of bending at different fluence rates. The experiments comprised 15 replicates. Data have been reprocessed from [17]. Two experimental protocols were used, called **PROT1** and **PROT2** here.

**PROT1** The coleoptiles were tilted at different initial angles *A*_0_, while the light was always maintained perpendicular to the initial orientation of the organ, *A*_*P*_ = *A*_0_ + *π*/2. The dependency of the *PGEA* on the fluence rate of the incident light *I* was then assessed by varying *I* at each value of the initial angle *A*_0_. *PGEA* can be plotted as a function of *I* for the different values of *A*_0_ (and the corresponding *A*_*P*_ = *A*_0_ + *π*/2).

**PROT2** The position of the *PGEA* was also measured for different values of *A*_0_, where *A*_*P*_ = *A*_0_ + *π*/2. However, the fluence rate of the incident light *I* was tuned experimentally so that the angle of the straight organ was immediately steadied at *A*_*R*_ = *A*_0_ stated in terms of the *A_R_C* model,. This provides an estimate of the value of *I*(*A*_*R*_ = *A*_0_) at photogravitropic equilibrium. The results of both protocols can be used to independently assess the $A_{R}^{a}(I)C$ model.

**Figure 2:**
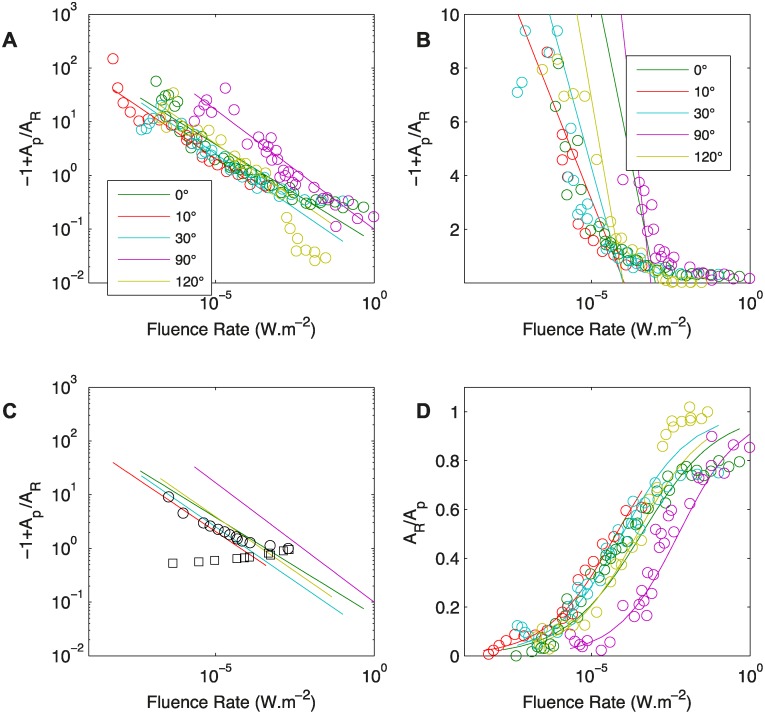
A. −1 + *A*_*P*_/*A*_*R*_ as a function of the fluence rate of the light. Data are reprocessed from Figs. 4, 5 and 6 in [17] Solid lines correspond to the fit $log(-1+\frac{A_{P}}{A_{R}})=a^{\prime }+b^{\prime }\log(I)$. An etiolated *Avena* coleoptile is tilted from the vertical for different values of the angle *A*_0_ (0, 10, 30, 90 and 120) while a light beam is collimated on the tip of the coleoptile. For different fluence rates, controlled by a neutral density filter, the apical angle is measured after variable duration. This is said to be the equilibrium angle. B. −1 + *A*_*P*_/*A*_*R*_ as a function of the fluence rate of the light; data are reprocessed from Figs. 4, 5 and 6 of [17]. Solid lines correspond to the fit $-1+\frac{A_{p}}{A_{R}}=a^{\prime }+b^{\prime }\log(I)$. C. −1 + *A*_*p*_/*A*_*R*_ as a function of the fluence rate of the light; data are reprocessed from Figure 8 in in [17]. The solid lines correspond to the fits from A. The empty black symbols (circles and squares) correspond to the measured fluence rates that precisely compensate the gravitropic reaction. The equilibrium state then corresponds to the case in which no movement is observed and *A*_*R*_ = *A*_0_. Empty circles: *A*_0_<90°; empty squares: *A*_0_>90°. D. *A*_*p*_/*A*_*R*_ as a function of the fluence rate of the light. Solid lines correspond to the fits from A.

The data produced in [17] according to the two protocols have been reprocessed. Linear orthogonal fit was carried out in a log-log plot and in a semi-log plot. A straight line in a log-log plot (as in Fig. 2.A) would reflect a power law, whereas a linear fit in a semi-log plot (as in Fig. 2.B) would reflect a logarithmic relation.

## Results

First, a simple experiment on wheat coleoptile with lateral light (LLE) is presented to get a rough estimate of the expected behavior during combined photo- and gravi-tropism movements.

Then to get a better estimate of the validity of the photogravitropic model, measurements of the apical angle of coleoptiles [17]) are compared with the prediction of the model. Besides providing a good agreement with the *A*_*R*_ *C* model, this agreement is only made for one specific organ. The different models are providing a more richer diversity that needs to be discussed.

The effect of each parameter in the model needs to be discerned as well as the respective influence of local and apical perception. To this end, the dynamics of the model of increasing complexity are presented.

Finally to obtain a full understanding of the model $A_{R}^{a}C$, it is proposed to study the different limits of the model. When one parameter is negligible or is dominating the dynamic, the specific effect of each parameter is accentuated. A deep, qualitative understanding of a complex system can then easily be reached.

The steady states of the different models are summarized in Table 3.

**Table 3:** Steady states of the different models.

*notations*	*steadystate*
*AC*	*A*(*s*) = *A*_0_ *e* ^−*βs*/*γ*^
*A* ^*a*^ *C*	$A(s)=A_{0}\left(1-\frac{s}{L}\frac{1}{1+\frac{\gamma }{\nu L}}\left(1-e^{-\left(\nu L\;+\;\gamma \right)t}\right)\right)$
*A*_*R*_ *C*	$A(s)=(A_{0}-A_{R})e^{-(\beta \;+\;\nu )s/\gamma }+A_{R}$
$A_{R}^{a}C$	$A(s)=(A_{0}-A_{R})\left(e^{-Bs/L}-e^{-B}\frac{1-e^{-Bs/L}}{M+\left(1-e^{-B}\right)}\right)+A_{R}$

The steady state, $\frac{\partial C}{\partial t}=0$, of the different models.

### Experimental assessment of the *A_R_C* model

A major prediction of the of *A*_*R*_-based models is that even when photoception and graviception act in different directions (i.e., *A*_*P*_ ≠ 0), they seem to act together to bring the organ towards a single direction defined by the angle *A*_*R*_. Additionally the *A_R_C* model predicts that the whole coleoptile curves in the direction *A*_*R*_ which is first achieved at the tip and the curvature then concentrates near the base.These two predictions are independent of the position at which light is perceived, i.e., at the tip of the organ or along the entire organ, and is thus common to all the variants of the *A*_*R*_ *C* model, (*i.e.* the local photoception model *A*_*R*_ *C* and apical photoception model $A_{R}^{a}C$ and their versions including explicit light intensity effects *A_R_*(*I*)*C* and $A_{R}^{a}(I)C$). For the sake of simplicity, whenever this common core is addressed, the notation *A*_*R*_ *C* will be used to refer to all of them.

**Figure 3:**
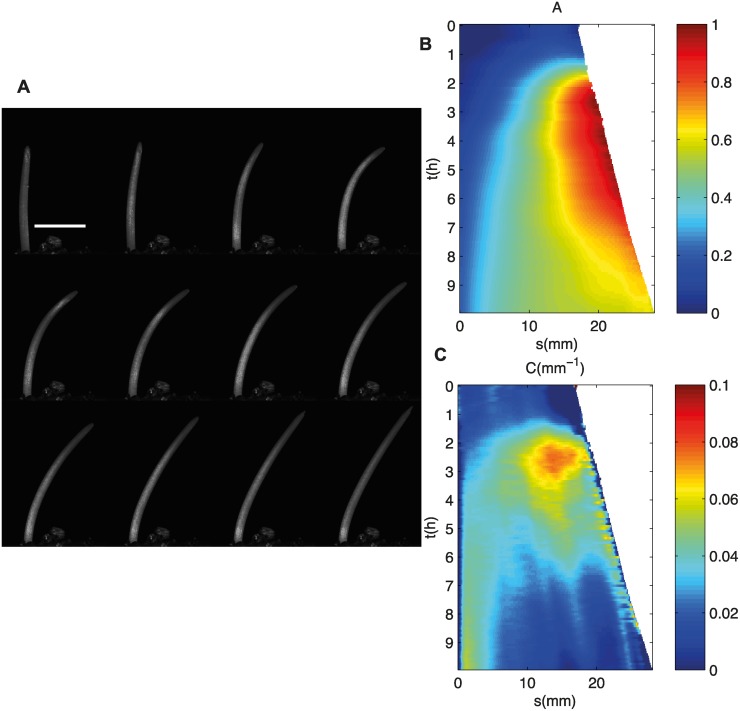
A. Timelapse of a coleoptile during phototropic movement, 50 minutes between each picture. The source light is collimated on the right of the organ and is perpendicular to the vertical. The white bar is 1*cm*. B. The kinematics of the angle *A*(*s*, *t*) plotted with respect to time *t* and curvilinear abscissa *s* (from the base to the apex of the organ). C. The kinematics of the curvature *C*(*s*, *t*) plotted with respect to time *t* and curvilinear abscissa *s* (from the base to the apex of the organ). At first, the whole organ curves; then the curvature concentrates near the base.

When illuminated by a source of light from a direction that differed from that of gravity (LLE experiment), the coleoptile curved and finally reached a steady state. The apical part aligned with an angle that was an intermediary between the direction of gravity (*A* = 0) and the direction of the light (*A* = *π*/2) (Fig. 3.A and B). This shows qualitative agreement with the *A_R_C* model. Additionnally the transient kinematic pattern in which i) the whole coleoptile curves until the direction *A*_*R*_ is first achieved at the tip and ii) the curvature then concentrates near the base. is qualitatively observed in (Fig. 3.B and C). However, this kinematic experiment also revealed some limits of grass coleoptile as a “model system” for tropic studies. Indeed, when a coleoptile is illuminated, photomorphogenetic effects take place, slowing down the expansion of the coleoptile and increasing that of the inner leaf. After approximately 10*h*, the leaf inside the coleoptile pierces the coleoptile. The dynamics of the coleoptile then ceases, and there is no further elongation of coleoptile. The dynamics of the movement is then fully determined by the leaf that was inside and this dynamics differs from that of the coleoptile. Moreover the influence of the inner leaf may explain why the rather steady tip angle (between 3 and 6 h in the example Fig. 3.A and B) is subsequently varied again (between 7 and 10h), and it is thus difficult to determine whether the coleoptile has had time to reach a steady state before been disrupted. However, the apical tip is predicted in the model to converge to the direction defined by *A*_*R*_ before the shape reaches a steady state, and we saw that this is consistent with what is observed experimentally. We may thus assume that in Galland [17] estimate of *A*_*R*_ could be measured even when the full steady state of the rest of the coleoptile could not be well defined.

We therefore tested further the validity of the functional form of *A*_*R*_-based models through the analysis of the apical tip angle at PhotoGravitropic Equilibrium (*PGEA*) in the work by Galland [17]. If the *A*_*R*_ *C* core-model is correct, then the experimental *PGEA* should be equal to *A*_*R*_, and according to equation 14, it should depend on the angle between the direction of gravity and direction of the light *A*_*P*_, and on the ratio between the gravisensitivity and the photosensitivity levels.

Indeed *A*_*R*_ can be expressed in the model as a function the photograviceptive number *M*:

$$A_{R}=A_{P}\frac{1}{1+M}$$ (25)

**Figure 4:**
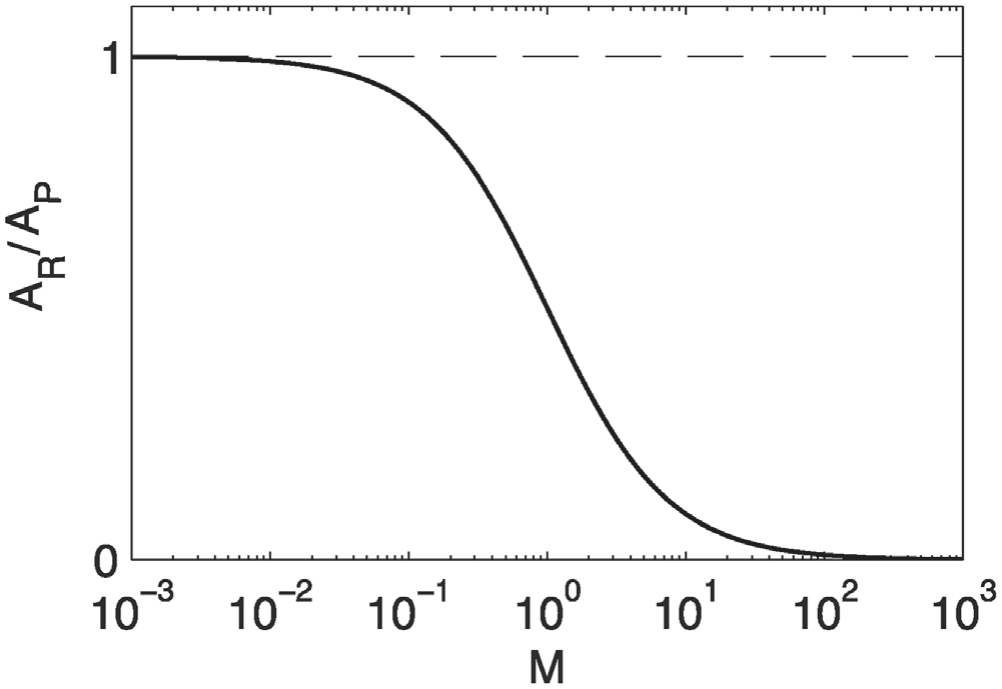
Variation of the reference orientation *A*_*R*_ as a function of *M*. When photoception dominates, *M* << 1, *A*_*R*_ = *A*_*P*_. When graviception dominates, *M* >> 1, *A*_*R*_ = 0.

It follows directly that when photoception dominates, the organ bends in the direction of the light, *M* < < 1, *A*_*R*_ = *A*_*P*_. However when graviception dominates, *M* > > 1, *A*_*R*_ = 0 (Fig. 4). It is then possible to express *M* directly as a function of *A*_*R*_/*A*_*P*_.

$$\begin{array}{c} M=\frac{A_{P}}{A_{R}}-1 \end{array}$$ (26)

A direct quantitative assessment of the functionnal form of the *A_R_*(*I*)*C* and $A_{R}^{a}(I)C$ models—and of the competing *M* ∼ Φ(*I*) sub-models—vs. experimental data can then be conducted through the analysis of the experimental relation between the steady-state tip angle *PGEA* and the fluence rate of the incident light *I*. Indeed *A*_*P*_ is known, and we have seen that we may assume *A*_*R*_ = *PGEA*. Then using equation 27 an estimate of M can be obtained. And combining equation 27 with equation 23, a prediction of the model is then that a plot of *M* = *A*_*P*_/*A*_*R*_ − 1 as a function of *I* should display either a power-law dependancy, equation 24, or a log-dependancy, equation 25. Moreover, these relations should fit to a single curve *M* ∼ Φ(*I*), independent of the initial tilting angle *A*_0_. Note however that due to the linearization of the sine terms of the angle dependency of gravi- and photoception (equations 1 and 4) this should be only valid for inclination angles *A*_0_ < 90°. Fig. 2 shows the compiled data from the first experimental protocol (**PROT1**) [17], reprocessed for comparison with the predictions of equation 27. The results of fitting equations 24 and 25 to the experimental data are also shown in Fig. 2 A and B. And Table 4 provides the values of the coefficients *a* and *b* of the curves fitting the power law (equation 24) for each initial angle *A*_0_. In the log-log plot in Fig. 2.A, the results of each individual experiment at a given *A*_0_ fit well to a straight line *M* ∼ *I* ^−*b*^ with values of *b* between 0.36 and 0.44 (Table 4). Except in cases in which *A*_0_>90°, the coleoptile response is independent of the initial angle *A*_0_, as predicted by the *A_R_C* model (in Fig. 2.A), and these data collapse on a single master curve. On the semi-log plot, however, no convincing fit can be identified. The power law relationship therefore seems to better describe the results than the logarithmic relationship does. Under the power law dependency of *M* on light intensity *I*, the coefficient of determination was *R*2 ∼ 0.91, such that our model captured 91% of the total changes in the steady-state tip angle attributable to changes in the initial angle *A*_0_ and fluence rate *I*.

**Table 4:** Parameters of the fit $log(1-\frac{A_{P}}{A_{R}})=a^{\prime }+b^{\prime }\log(I)$.

*A*_0_	*a* ^′^	*b* ^′^	*R* ^2^
0	18	0.36	0.88
10	47	0.40	0.95
30	43	0.41	0.94
90	10	0.44	0.72
120	28	0.41	0.89

Value of the parameters *a* ^′^ and *b* ^′^ given by the fit $log(1-\frac{A_{P}}{A_{R}})=a^{\prime }+b^{\prime }\log(I)$ for different values of the initial angle *A*_0_ of the plants (Fig. 2A).

The response for higher angles does not follow the first order relation expressed in the *AC* model anymore, but the sine laws. It is then expected that, for inclination angles *A*_0_ > 90°, the experimental data are adrift from this fit and vary non-monotonously, as *A* varies in a way that can no longer be accounted for by the *A_R_C* model. This is indeed observed in Fig. 2.

The second protocol (**PROT2**) provides a different way to measure the same parameter than **PROT1**, even if the protocol is different. The plot of *A*_*R*_/*A*_*P*_ = *A*_0_/*A*_*P*_ as a function of the experimental values of *I* should then collapse on the single master curve defined with **PROT1** in Fig. 2.C. Again, the model predictions were fair at small angles, *A*_*R*_/*A*_*P*_ ≤ 0.4, but for larger angles the experimental data diverged non-monotonously from the model. It is interesting to note that the results fit well to both the logarithmic law and the power law [17].

Finally a last testable prediction of the *A_R_C* model is that the plot of *A*_*P*_/*A*_*R*_ as a function of *M*, and hence of the light fluence rate *I*, should be S-shaped with two asymptotes: i) when the light intensity is low, gravity dominates and *A*_*R*_ = 0 and ii) when the light intensity is high. Moreover the equilibrium *A*_*R*_ is shifted toward the orientation of incident light such that *A*_*R*_ = *A*_*P*_ (Fig. 4). These predictions are also consistent with the experimental data shown in Fig. 2.D.

Now that experimental agreement has been found with features of the photogravitropic models, it is interesting to discuss more in details the properties of the models and the insights it provides on the photo- and gravi-tropic control. This will be conducted through considering variant models of increasing complexity.

### Insights on phototropism brought by the models: Photoception and proprioception

#### Apical photoception: *A^a^C* model

**Figure 5:**
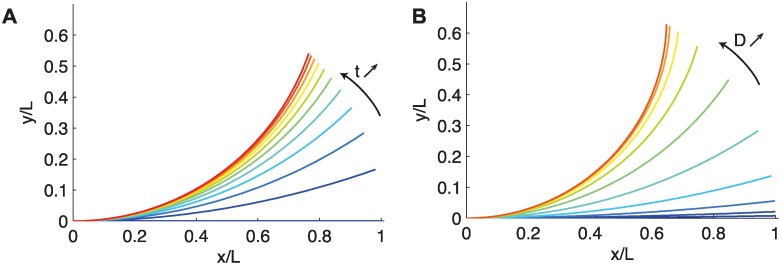
A. Straightening dynamics of the *A_a_C* model (*D* = 4). At each time the shape is an arc of circle. B. Steady-state shape of the *A^a^C* model. The shape is an arc of circle. As photoception dominates over proprioception, *D* increases (from blue to red); the apical angle reorients in the direction of the light field vector.

When photoception is only apical (the *A^a^C* model; equation 7), the curvature is constant along the organ (no dependency on *s*), so the shape of the organ is an arc of a circle (Fig. 5.A). This can be understood as the organ trying to bring the apical part towards the vertical with an identical driving signal everywhere. Finally, at the steady state, the curvature is also constant along the organ so that the orientation *A*(*s*) changes linearly from the base to the tip, where it reaches the vertical (Fig. 5.B). The solution of equation 7 depends only on the photoproprioceptive number *D* and on the propriosensitivity *γ*, but in a contrasted way.

The apical angle of the steady state of the *A^a^* model is given by

$$A\left(L,t\to \infty \right)=A_{0}\frac{1}{1+D}$$ (27)

and the curvature of the steady state is simply defined by

$$C\left(t\to \infty \right)=A_{0}L^{-1}\frac{1}{1+D^{-1}}.$$ (28)

The apical angle and the curvature at the steady state are thus direct indicators of the ratio *D* between photoception and proprioception. When the photoceptive term dominates over the proprioceptive term (*D* → ∞), the apical angle tends to approach the direction of the light (Fig. 5.B). In sharp contrast to what was found for gravitropism [2], when apical perception is involved, proprioception is not necessary to reach a steady state. If *γ* = 0, the movement stops when the apical part is parallel to the direction of the light. Consequently (and concurrently), all parts of the organ near the base also cease to curve. Proprioception only modifies the maximal curvature that the system can reach. When proprioception increases (i.e., *D* decreases) the apical angle at steady state no longer reaches the vertical. In any case, however, the apical perception shapes the organ as an arc of a circle.

Considering now the transient dynamics before convergence to the steady state, the characteristic time at which the apical part of the organ reaches the vertical is given by

$$T_{c}=\gamma^{-1}\frac{1}{1+D}=\frac{1}{\gamma +\nu }$$ (29)

Note that this characteristic time depends on both the proprioceptive and the photoceptive terms. This differs from the case in the *AC* model [2], in which, due to the purely local control, the characteristic time depends only on the proprioceptive term. The inclusion of a non-local photoceptive term thus makes the organ converge to its steady state faster than in the case of purely local perception. Also in contrast to the case of gravitropic movement [2], the dynamics of phototropic movement, governed by photoception and proprioception, cannot not be fully described by a unique dimensionless number, such as in this case *D*.

#### Local photoception: Back to the *AC* model

In the case in which light perception is local, the photoproprioceptive model takes the form of an *AC* model (equation 11). The dynamics and the steady- state shape are thus fully described by the dimensionless number, in this case *D*, alone. The salient features of the movement can then be summarized as follows: i) Initially the whole part of the plant that responds to the stimuli starts curving; ii) then, the curvature concentrates near the base while the apical part straightens; and iii) the number of oscillations around the vertical before convergence to the steady state increases with *D*. The dimensionless number *D* also reflects the ratio between the length of the organ and the length of the curved zone at steady state, in a strict analogy with what was found for the dimensionless number *B* in the *AC* model for gravitropism [2]. And in this case proprioception is necessary to avoid infinite oscillations and reach a steady state.

Having established a clear understanding of the behavior under the sole influence of either phototropism or gravitropism [2], we can now discuss the interaction between phototropism and gravitropism.

### Insights on photo-gravitropic interaction brought by the models

In order to reach insights on the dynamic of the interactions between photo -, gravi- (and proprio-)-ceptions on the control of the tropic movement, we will consider the case of a tropic motion at constant incident light intensity *I*, therefore studying the *A_R_C* model (the influence of *I* through changes in *M*, the ratio between photoception and graviception, having been clarifyied previously).

#### Local photoception: the *A_R_C* model

The dynamics of the *A_R_C* model (equation 15), where graviception and photoception both occur locally, is strictly equivalent to the dynamics of the graviproprioceptive*AC* model [2]. It is thus controlled by a unique dimensionless number. This dimensionless number drives the shape of the steady state (for example, the length *L*_*c*_ over which the organ is curved at steady state), as well as the number of transient oscillations before the steady state is reached. But there are some differences between the two models: the dimensionless number, denoted *B* ^′^, that drives the full dynamics of the *A_R_C* model is a composition of the graviproprioceptive number $B=\frac{\beta L}{\gamma }$ and of the photoproprioceptive number *D*

$$B^{\prime }=\frac{(\beta +\nu )L}{\gamma }=B+D$$ (30)

As *D* ≥ 0, the dimensionless number *B* ^′^ is larger than the graviproprioceptive number *B*, *B* ^′^ ≥ *B*. The convergence length *L*_*c*_ is then modified

$$L_{c}=\frac{L}{B^{\prime }}$$ (31)

This involves quantitative differences in the dynamics of the *A_R_C* model compared to that of the graviproprioceptive *AC* model. As *B* ^′^ is larger than *B* (*D* ≥ 0), the tropic movement driven by the local photograviproprioceptive *A_R_C* model (equation 12) exhibits more transient oscillations than does the graviproprioceptive tropic movement driven by the *AC* model (for the same *B*), as well a smaller convergence length *L*_*c*_.

In this case we can demonstrate that the tip is converging to the resultant photogravitropic angle *A*_*R*_. Indeed, according to equation 15, the apical angle of the steady state of the *A_R_C* model is given by

$$A^{\prime }(L,\;t\to \infty )\;=\;{A^{\prime }}_{0}e^{-B^{\prime }}={A^{\prime }}_{0}e^{-(B+D)}$$ (32)

$$A(L,\;t\to \infty )\;=\;(A_{0}-A_{R})e^{-(B+D)}+A_{R}$$ (33)

In the absence of light, *D* = 0, if the apical part of the organ reaches the vertical, *A*(*L*, *t* → ∞) ∼ 0, it is expected that *e* ^−*B*^ ∼ 0. As the length of convergence is smaller when phototropism and gravitropism interact than in the gravitropic case, *B*+*D*>*B*, the term *e* ^−(*B* + *D*)^ can therefore be neglected

$$A\left(L,t\to \infty \right)∼A_{R}$$ (34)

Just as for *B* in the gravitropism case [2], it then becomes possible to design a simple morphometric estimate of *M* (the ratio between photoception and graviception at the current incident light intensity) for a plant organ with local and distributed gravisensitivity and photosensitivity, such as the *Arabidopsis* hypocotyl. The measurement of the apical angle of the steady state provides a simple way to directly measure *A*_*R*_ and the ratio between photoception and graviception *M* can then be readily estimated using equation 27.

#### Apical photoception: $A_{R}^{a}C$ model

The $A_{R}^{a}C$ model (equation 19), in which photoception is apical and graviception is local (as in the case of grass coleoptiles), is now explored. The steady-state shape of the $A_{R}^{a}C$ model is given by equation 21. The left term inside the brackets of the steady-state shape defined by *A* ^′^(*s*, *t* → ∞) (equation 21) is the steady-state shape of the graviproprioceptive solution, the *AC* model, alone

$$A_{0}^{\prime }e^{-Bs/L}$$ (35)

whereas the right term can be considered as a corrective term resulting from the interaction between photoception and graviception.

$$A_{0}^{\prime }e^{-B}\frac{1-e^{-Bs/L}}{M+\left(1-e^{-B}\right)}$$ (36)

This $A_{R}^{a}C$ model shares some properties with the *A_R_C* model. The perceptions of light and gravity act together to bring the system in the direction *A*_*R*_, which results from the combination of the two different tropisms. But unlike the empirical equation for the photogravitropic equilibrium *PGEA* assumed by [17] (equation 2), the angle *A*_*R*_ defined through the equation 26 does not depend on the addition of the two stimuli but on the ratio between the two sensitivities, the photograviceptive number *M*. The length of the curved zone, *L*_*c*_, depends only on the graviproprioceptive term *B*, $L_{c}=\frac{\gamma }{\beta }=\frac{L}{B}$. However, in contrast to the case in which graviception is dominated by proprioception, *L*_*c*_ → ∞, the curvature is not necessarily equal to 0 (see equation 22) and depends on the value of the photograviceptive number *M*.

Finally, it is still possible to design a simple morphometric estimate of *A*_*R*_, of the ratio between photosensitivity and gravisensitivity *M* and of the ratio between gravisensitivity and propriosensitivity *B*. But this now requires the combination of two experiments, one in the light, and one in the dark. As in the *A_R_C* model, the measurement of the apical angle of the steady state in a photogravitropic experiment provides a simple way to directly measure *A*_*R*_ (this will be further investigated when studying the limit cases of the $A_{R}^{a}C$ model in the next section). *B* can then be estimated independently using a gravitropic tilting experiment in the dark on the same organ (as in [2]). Finally the ratio between photosensitivity and gravisensitivity *M* can be calculated using the steady-state (equation 21) at *s* = *L*.

#### Limit cases and sensitivity analysis of the $A_{R}^{a}C$ model

The behavior of the $A_{R}^{a}C$ model is complex. To obtain a better understanding of it, in this section we explore the limit cases. The model’s dynamics can be described using only *B*, the ratio between graviception and proprioception, and *M*, the ratio between photoception and graviception. Therefore only four different limit cases are to be considered: *B* → ∞, *B* → 0, *M* → ∞, *M* → 0. In what follows we characterize each case. Moreover, starting from each given limit case, we gather insights regarding the control dynamics by varying one of the dimensional numbers. This essentially constitutes an analysis of sensitivity of the output of the photo-gravi-proprioceptive model at steady state to changes in its dimensionless control parameters *B* and *M*.

i) *B* → ∞. Graviception dominates proprioception. This case has already been shown to be a non-physiological case [2]. Indeed, in this case there is no steady state. The organ cannot converge to the vertical and oscillates indefinitely, displaying no tropism.

ii) *B* → 0. Proprioception dominates graviception. *A*_*R*_ is given by the direction of the light *A*_*R*_ = *A*_*P*_. The final shape is then given by

$$A^{\prime }\left(s\right)=A_{0}^{\prime }\left(1-\frac{s}{1+D^{-1}}\right)$$ (37)

This is the solution of the *A^a^C* model, equation 3.

**Figure 6:**
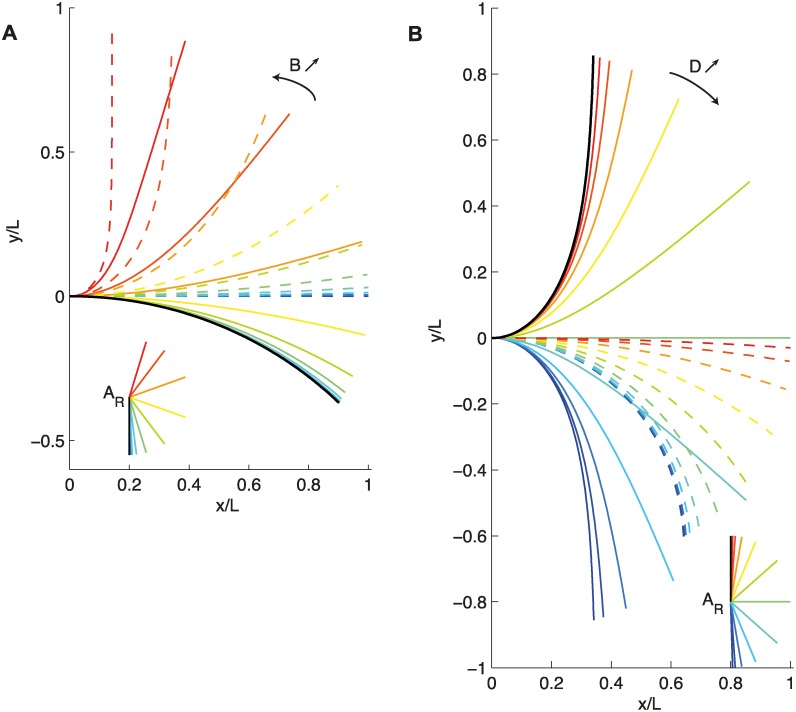
A. Steady-state shape of the $A_{R}^{a}C$ model for D = 4. The black line is the solution when *B* = 0. As *B* increases (solid colored line from red to blue), the orientation and the shape of the organ is modified. The dashed line shows the steady-state shape of the graviceptive equation. The expected orientation of the organ *A*_*R*_ is shown at the bottom. B. Steady-state shape of the $A_{R}^{a}C$ model for *B* = 4. The black line is the solution when *D* = 0. As *D* increases (solid colored line from red to blue), the organ’s orientation and shape are modified. The dashed line shows the steady-state shape of the photoceptive equation, *A^a^C* model. The expected orientation of the organ *A*_*R*_ is shown at the bottom.

Starting from this limit case of pure photoproprioceptive equilibrium, it is now useful to consider the changes of the steady-states of the photograviproprioceptive model that occur when the graviproprioceptive number *B* is increased from 0 while the photoproprioceptive number *D* is maintained at a constant value (Fig. 6.A). This corresponds to increasing the gravisensitivity *β* relative to the photosensitivity *ν*, and hence to increasing the photograviceptive number *M*. As this change first affects the graviproprioceptive part of the $A_{R}^{a}C$ model, the changes in steady state of the graviproprioceptive *AC* model with increasing *B* are also plotted for comparison (dashed lines in Fig. 6.A). Starting from the arc-shaped solution of the photoproprioceptive *A^a^C* model, increasing *B* (and *M*) for a constant *D* brings the photo-gravi-proprioceptive model into steady states in which the apical part orients toward the changing *A*_*R*_ (see insert in Fig. 6.A), whereas the curved zone becomes more and more concentrated at the base. Note also that the solution for the photo-gravi-proprioceptive $A_{R}^{a}C$ model is no longer equivalent to a rotation of the graviproprioceptive *AC* model toward *A*_*R*_, but it is striking that the alignment of the apical part toward *A*_*R*_ is maintained and actually increases with *B*.

iii) *M* → ∞. Graviception dominates photoception. For a given graviproprioceptive ratio *B*, this also means a very low photoproprioceptive number *D*, i.e., *D* → 0. The direction of the expected orientation is the direction of gravity, *A*_*R*_ = 0. It follows easily that the steady state is given by:

$$A\left(s\right)=A_{0}e^{-Bs/L}$$ (38)

This is the solution of the graviproprioceptive equation, the *AC* model, equation 1.

We may now study the effect of increasing photosensitivity, i.e., increasing the photo-graviceptive number *D* and, by necessity, decreasing the photograviceptive number *M* (Fig. 6.B) for a constant value of *B*. As this change is first affecting the photoproprioceptive part of the model, the changes in the steady state of the photoproprioceptive *A^a^C* model with increasing *D* are also plotted for comparison (dashed lines in Fig. 6.B). When increasing *D* (and decreasing *M*) at constant *B*, changes in the steady-state shapes of the photo-gravi-proprioceptive $A_{R}^{a}C$ model are driven by changes in *A*_*R*_ (Fig. 6.B). However, the solution never converges toward the photoproprioceptive solution of the *A* ^*a*^ *C* model. The orientation of the stem changes as *D* changes, but the length of the curved zone remains the same (as *B* is fixed). Even if graviception and photoception contribute equally towards orienting the apical part, they play different roles in the control of the global shape of the organ.

iv) *M* → 0. Photoception dominates graviception. In this case, the expected orientation is driven simply by the direction of the light, *A*_*R*_ = *A*_*P*_. The steady-state shape is then given by

$$A^{\prime }(s)\;=\;A_{0}\left(e^{-\frac{Bs}{L}}-\frac{1-e^{-Bs/L}}{e^{B}-1}\right)$$ (39)

$$C(s)\;=\;\frac{BA_{0}}{L}\frac{e^{-\frac{Bs}{L}}}{1-e^{-B}}$$ (40)

It should be noted that, for any *B* that is not equal to zero, this solution is not the photo-proprioceptive *A^a^C* model but rather is a modification of the graviproprioceptive *AC* model (compare the plain line and the dashed lines in Fig. 6.B, especially for high values of *D*; green and blue-colored curves). The photoception sets the apical angle

$$A^{\prime }\left(s\right)=0$$ (41)

but the global shape is just a perturbation of the graviproprioceptive shape of the *AC* model. So even if photoception dominates over graviception, if the graviception does not tending towards *B* = 0, the distribution of the curvature in the steady state is still clearly determined by graviception and proprioception and thus by *B*.

**Figure 7:**
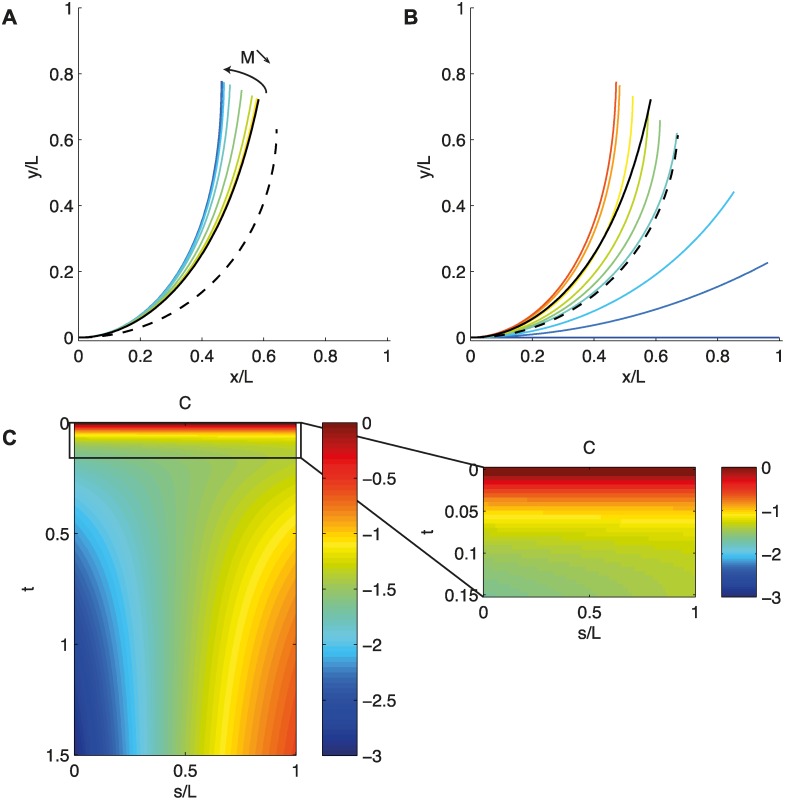
A. Steady-state shape of the $A_{R}^{a}C$ model for *B* = 2. The solid black line is the solution when *D* = 0; the dashed line is the solution when *D* → ∞ and *B* = 0. As *M* increases (from red to blue), the organ’s orientation and shape are modified. B. Straightening dynamics of the $A_{R}^{a}C$ model (*B* = 2 *D* = 20). The solid black line is the solution when *D* = 0; the dashed line is the solution when *D* → ∞ and *B* = 0. During the movement, the organ reaches the steady state of the photoceptive equation but then goes to the steady state of the gravi-photo-proprioceptive equation. C. Space-time mapping of the curvature *C*(*s*, *t*) during straightening.

The complete mechanism of control over the steady-state shape in the case of photogravitropism can now be summarized. The orientation of the apical part is determined by the ratio between graviception and photoception, the photograviceptive number *M*, whereas the length of the curved zone is determined only by the ratio between graviception and proprioception, the dimensionless number *B*. This can be illustrated clearly by considering the case in which the two tropisms act in the same direction (*A*_*P*_ = 0, Fig. 7.A). As in previous examples, the initial condition is *D* = 0 (pure graviproprioceptive steady state) but with a lower value of *B* (so that proprioception prevents the tip from aligning in the direction of gravity). In these conditions, decreasing the value of *M* (by increasing *D* for a constant *B*) modifies the apical angle toward the vertical (phototropism reinforces gravitropism in this case), but the length of the curved zone remains unchanged (being determined solely by the graviproprioceptive process, and thus by the value of *B*).

It is possible to use a similar analysis procedure to investigate the dynamics of movement within the $A_{R}^{a}C$ model, before convergence to the steady state (*i.e.* shape of the transients and time to converge to a steady state).. The first cases are straightforward.

i) *B* → ∞ Graviception dominates proprioception. The organ never reaches the vertical [2].

ii) *B* → 0 Proprioception dominates graviception. The dynamics are described by apical phototropism alone, *i.e.* the dynamics of the *A^a^C* model, as described in the corresponding section (Fig. 5).

iii) *M* → ∞ Graviception dominates photoception. The dynamics of the $A_{R}^{a}C$ model are equivalent to the dynamics of the graviproprioceptive *AC* model as described in [2]. Briefly, for higher values of *B* (i.e., greater dominance of graviception over proprioception), the transients are more contorted, and the organ oscillates for a longer time around the steady state before converging.

iv) *M* → 0 Photoception dominates graviception. This case must be discussed more carefully. In this case, the two processes, graviception and photoception, act as dominant drivers at two different time scales. In the photo-gravi-proprioceptive $A_{R}^{a}C$ model, photosensitivity has a faster effect than gravisensitivity does. This can be illustrated clearly by considering again the case in which the two tropisms act in the same direction (*A*_*P*_ = 0, Fig. 7.B). The model predicts that the organ will bend faster towards the light in order to bring the apical part to the direction of the light, and to reach the steady-state shape of the photoproprioceptive equation. Then, the graviproprioceptive process takes over. The curvature concentrates near the base while the apical part remains oriented in the direction of *A*_*P*_ (Fig. 7.B and C). This behavior can be compared to the experiments of the Fig. 3. First, the whole coleoptile is curving and the apical tip reaches an angle bigger the angle at steady state. Then the curvature concentrates near the base as the apical straightens. Similarities between the theoritical behavior and the experimental behavior are then observed. However due in part to the specific dynamics of the coleoptile and the inner leaf, it seems difficult to discuss further away the similarities and disparities between experiments and theory.

## Discussion

This study shows that a minimal modeling approach used to study shoot gravitropism, which gave rise to the *AC* model [2], can be readily extended to study the interactions among photoception, graviception and proprioception. The extended model, the *A_R_C* model, is minimal but complete. Simple dimensionless control parameters can be estimated from the *A_R_C* model, and the model is tractable and easy to understand. Furthermore, it is possible and straightforward to include the dependency of photosensitivity on light intensity *I*, thereby producing an augmented *A_R_C* model. Predictions from the latter model were assessed against detailed quantitative experimental results [17]. The model agreed very well with the data for small initial angles. All the experimental curves at various inclination angles and light intensities collapse to a single curve when using the *A_R_C* model with a power-law dependency between the photograviceptive control number *M* and the intensity of light *I*. Although the assessment is not comprehensive, the evidence presented supports the validity of the model. The hypotheses that the action of the tropic motor is fully driven by perception-regulation processes (H1), that the angles between the gravity and the light fields are first-order variables influencing the tropic dynamical movement (H2), that the two types of perceptions act additively (H4), and that the balance between gravisensing and photosensing depends on the perception of light intensity (H5) are upheld in the model.

The simplified versions and limiting cases of the *A_R_C* model served as useful tools for analyzing the dynamical interactions associated with perception during tropic movement, yielding five major insights.

i) Despite having different set-points, gravitropism and phototropism act together to align the organ with the direction defined by *A*_*R*_ (equation 26). *A*_*R*_ is determined by the dimensionless number *M* and hence by the ratio between gravisensitivity and photosensitivity. As in the *AC* model, the tropic movement is controlled globally through the different types of tropic perception, which together drive the local curving velocity. The final steady-state shape reflects the ratio of the respective sensitivities to light and gravity [2].

This may seem to be at odds with the phenomenological model for *PGEA* proposed by Galland, summarized in equation 2 [17], which states that the global steady-state shape at photo-gravitropic equilibrium depends additively on the phototropic and gravitropic stimuli, not on their ratio. However this phenomenological model for *PGEA* becomes problematic when considering the limiting cases. According to equation 2, in darkness (i.e., when *I* = 0) the *PGEA* = −*g* sin(*A*_0_). In other words, the gravitropic equilibrium orientation is a function of the initial angle. However, when there is no light, the set-point becomes simply the gravitropic set-point angle (*GSA*) [28], and the equilibrium shape is driven by the *GSA* and by the graviproprioceptive number *B* [2]. For ortho-gravitropic organs (i.e., in which the *GSA* is vertical) in plants in which graviception dominates proprioception (where the value of *B* is large), experimental observations confirm that the equilibrium tip angle is indeed vertical, that is, *PGEA* = 0 for any initial angle *A*_0_ [2, 22]. The final gravitropic steady-state shape does not depend on sin(*A*_0_) at all. Rather, the sine law dependency applies to the transient rate of changes in curvature, not to the final steady-state shape [1, 2, 4, 34]. In the other limiting case, when photoception dominates gravisensing (e.g., when the light is very bright, in microgravity or in agravitropic mutants), the set-point angle is determined by the direction of the light alone. The steady-state tip angle should therefore tend to *A*_*P*_. The expression of the *A*_*R*_ in equation 26 accommodates this condition, whereas the phenomenological model for *PGEA* in equation 2 predicts a tip angle that would depend on the light intensity with no saturation. The phenomenological model for *PGEA* [17] is thus not consistent for these two limiting cases.

The expression of the *A*_*R*_ as the ratio between the sensitivities overcomes these problems directly and is also consistent with a complete dynamical model, the *A_R_C* model. *A*_*R*_ is thus a better indicator than the previously defined *PGEA* [17]. In order to to unify the notation, we propose to follow [26], and to refer to this angle *A*_*R*_ as the PhotoGravi Set Point Angle (*PGSA*).

ii) The relation between the intensity of light (*I*) and the tropic response is better described by a power law, *ν* ∼ *I* ^−*b*^, than by a logarithmic law [17]. The power-law relationship was found to be more robust, fitting all the different experiments over more orders of magnitude of light intensity. For small angles, it was possible to collapse the different experimental results at various inclination angles and various light intensities into a single master curve. As the power law, the log law only relies on two parameters, however the log law also requires the assumption of a threshold in the perception of light. The power law is then more parsimonious. Further studies should be performed to investigate the photochemical basis of such an invariant scaling of the photo-gravisensitivity balance with light intensity, and to confirm the values of the exponent of the power law, estimated here as *a* ∼ 0.4.

iii) The respective effects of local distributed perception along the organ and of apical perception are clearly defined for the first time. The *A^a^C* model, which deals with purely apical perception, predicts that the shape formed by the responding organ is an arc of a circle. It is reasoned that all the cells located along the organ receive the same secondary signal from the apical sensory apparatus, so every part of the organ curves to the same extent in order to bring the apical part into the direction of light. This prediction could be assessed experimentally on aerial organs but would require agravisensitive experimental conditions in which the $A_{R}^{a}(I)C$ model can be reduced to the *A^a^C* model. Experimental conditions that approximate the absence of gravisensitivity may be achieved using microgravity, clinostat experiments [32, 41], or mutant genotypes with impaired gravisensing [30, 41]. The relevant control parameter is the dimensionless photoceptive number *D*, which measures the ratio between photoception and proprioception. *D* can be easily estimated by measuring the apical angle of the steady state and the basal angle *A*_0_, two variables routinely measured in most experiments on tropism.

iv) Local perception (gravitropism) and apical perception (in species with apical phototropic sensing) interact so that aerial organs can forage for light without losing mechanical stability. Indeed, according to the $A_{R}^{a}(I)C$ model, the plant strives to align with the direction set by the ratio between photoception and graviception, but the curvature concentrates near the base while the apical part straightens. The length of the curved zone is under graviproprioceptive control only. This could be a functional adaption: Although the shoot must grow toward the light, the posture of the stem also needs to be controlled to ensure long-term stability. This may also explain how the apical part of some plants such as sunflowers can track the sun daily, while the plants continue to maintain control over their posture.

v) Information from the *A_R_*(*I*)*C* model can be used to design methods for high-throughput phenotyping of the complete tropic control of organs by measuring the dimensionless parameters *B*, *D* and *M* of individual plants. *B* can be determined by measuring a plant’s entire shape in simple gravitropic experiments in the darkness [2]. *D* can technically be measured in non-gravisensing conditions. However, it is simpler and faster to directly estimate *M* from the *PGSA*. Indeed, *M* is the ratio between gravisensitivity and photosensitivity, so the experiment can be conducted in gravisensing conditions. The apical angle at the steady state is the only measurement needed in order to estimate *M*. Once *B* and *M* are known, the value of *D* can be readily calculated, and the entire set of parameters controlling the dynamics of the system is then quantified. The dimensionless numbers *B*, *D* and *M* are real quantitative genetic traits that can be used to phenotype tropic mutants in genetically amenable plant models [30, 41] and subsequently identify the genetic and molecular mechanisms controlling tropic movements in plants.

The main limitation of the *A_R_*(*I*)*C* model in its present form is that it only deals with a geometrically simple light field, i.e., with uniform light and with the gravity field **g**, where the plant and the light field **l** lie within the same plane. It is possible, for example, to create spherical light fields by setting up point sources of light in proximity to the plant. The model presented here can be extended to include this case by making the light sensitivity parameter *ν* dependent on the spatial position *ν*(**x**, t). It is likely that the geometry of light fields in nature is even more diverse. For example, the direction of the sun’s light changes over the course of the day. The direction of the light may also be outside the plane defined by the plants and the gravitational field. The modeling framework developed herein could be extended to deal with more complex light-field geometries, but this would require further mathematical and programming development. Such extensions would enable diverse movements to be simulated and would provide a more complete understanding of the ecological function of tropisms [5] beyond the core example of tropic control presented in this study.

It should also be noted that the use of etiolated organs (e.g., coleoptiles and hypocotyls) can complicate the analysis of phototropism. These organs are subject to photomorphogenetic effects that modify their behavior, independently of phototropic movements. Hypocotyl geometry is modified by the opening of the hook [42, 43]; in addition, it has been shown that pretreatment with light affects hypocotyl behavior [44]. Fully developed organs, which are less susceptible to photomorphogenetic effects, are therefore a powerful tool in the study of phototropism. Thus, kinematic analysis of the inflorescence of *Arabidopsis thaliana* might yield powerful insights regarding the phototropism process [19].

Finally and more generally, our modeling approach may also be used to study the tropic responses of other organisms, such as fungal stripes, sporangiophores or hyphae [18, 45], or of other plant organs such as roots. Plant roots might provide an interesting system in which to test the effects of apical perception and to assess the validity of the *A^a^C* model. As graviception in roots is purely apical [41], the *A^a^C* model can be readily extended to root gravitropism with the caveat that the extent of proprioception in roots remains unknown. This model could then be assessed by measuring the steady-state curvature (predicted to be constant along the organ) in hydroponics experiments to avoid confounding effects of interactions with substrates. If the model is validated, it may be possible to confirm a unified theory of the tropic movements of fixed organisms in natural conditions.

## Supporting Information

S1 Text Supplementary information, Analytical results of the models and modeling aspects concerning the effect of the time of signal propagation along the organ. Figure S1—Solution of the *A_a_C* model for instantaneous propagation, *T*_*B*_ = 0. Figure S2—Simulation of the *A_a_C* model for different value of the ratio *T*_*B*_/*T*_*C*_. As long as the the propagation is faster than the characteristic time of the movement, the solution is similar to the solution with instantaneous propagation. Figure S3—Orientation of the organ in the *AaC* model for different value of the ratio *T*_*B*_/*T*_*C*_.
(PDF)
