# Supplementary material for: A Unified Model of Shoot Tropism in Plants: Photo-, Gravi- and Propio-ception
Source: PLoS Comput Biol. 2015 Feb 18;11(2):e1004037. doi: 10.1371/journal.pcbi.1004037 (PMC4332863; doi:10.1371/journal.pcbi.1004037)
Supplement: S1 Text — Figure S1—Solution of the AaC model for instantaneous propagation, T B = 0. Figure S2—Simulation of the AaC model for different value of the ratio T B/T C. As long as the the propagation is faster than the characteristic time of the movement, the solution is similar to the solution with instantaneous propagation. Figure S3—Orientation of the organ in the AaC model for different value of the ratio T B/T C. (PDF) [file pcbi.1004037.s001.pdf]

A Unified Model of Shoot Tropism in plants: Photo-, Gravi- and Propio-Ception — Supplementary Material

Renaud Bastien[1,2,3,4], Stéphane Douady[5,*], Bruno Moulia[2,3,*]

Models - Analytical Results

$A_a C$ model

The apical photoceptive $A_a C$ model without graviception is defined by

$$\frac{\partial C(s,t)}{\partial t} = -\nu A(L,t) - \gamma C(s,t) \tag{1}$$

where s is the curvilinear abscissa along the organ, from the base $s = 0$ to the tip of the organ $s = L$ and t is the time. $\nu A(L,t)$ is the apical photoceptive term, readily transmitted all along the organ, and $\gamma C(s,t)$ is the proprioceptive term.

Let now the initial conditions be a straight but tilted organ and the boundary conditions of a basal perfect clamping, :

$$A(s,0) = A(0,t) = A_0 \qquad C(s,0) = 0 \tag{2}$$

It is easy to see that the $A_a C$ model is in fact independent of space coordinate s. As the perception of the light is purely apical, each part of the organ receives a signal proportional to the same apical orientation at any time. Furthermore with the initial conditions in which the organ is straight, the curvature remains the same everywhere. Then by definition of the curvature, it comes that

$$A(L,t) = A_0 + C(t)L \tag{3}$$

and the equation 1 could be rewritten

$$\frac{dC(t)}{dt} = -\nu A_0 - (\nu L + \gamma)C(t) \tag{4}$$

the general solution of this equation is given by the equation

$$\frac{dC(t)}{dt} + (\nu L + \gamma)C(t) = 0 \tag{5}$$

It is easily solved by integration

$$\int_{C(0)}^{C(t} \frac{dC'(t)}{C'(t)} = -\int_0^t (\nu L + \gamma) dt' \tag{6}$$

then

$$\log C(t) = -(\nu L + \gamma)t + K \tag{7}$$

where K is a constant, and finally

$$C(t) = Ke^{-(\nu L + \gamma)t} \tag{8}$$

The particular solution of the equation 4 is

$$C(t) = -\frac{\nu}{\nu L + \gamma} A_0 \tag{9}$$

so the solution is

$$C(t) = Ke^{-(\nu L + \gamma)t} - \frac{\nu}{\nu L + \gamma} A_0 \tag{10}$$

K must be identified with the initial conditions, $C(0) = 0$

$$K = \frac{\nu}{\nu L + \gamma} A_0 \tag{11}$$

yielding

$$C(s,t) = C(t) = -A_0 \frac{\nu}{\gamma + \nu L} \left(1 - e^{-(\nu L + \gamma)t}\right) \tag{12}$$

which can be rewritten with the dimensionless number D

$$C(s,t) = -\frac{A_0}{L} \frac{1}{1+D} \left(1 - e^{-\gamma(1+D)t}\right) \tag{13}$$

The orientation of the organ in the A_a model is then given by the integration along the organ

$$A(s,t) = \int_0^s C(s',t) ds \tag{14}$$

yielding

$$A(s,t) = A_0 \left(1 - \frac{s}{L} \frac{1}{1+D} \left(1 - e^{-\gamma(1+D)t}\right)\right) \tag{15}$$

$$\tag{16}$$

The apical angle of the steady state of the A_a model is given by

$$A(L, t \to \infty) = A_0 \frac{1}{1+D} \tag{17}$$

and the curvature of the steady state is simply defined by

$$C(t \to \infty) = A_0 L^{-1} \frac{1}{1+D^{-1}} \tag{18}$$

If there is no proprioception, $\gamma = 0$, the dynamics is defined by

$$A(s,t) = A_0 \left(1 - \frac{s}{L} \left(1 - e^{-\nu L t}\right)\right) \tag{19}$$

$$C(s,t) = A_0 \left(1 - e^{-\nu L t}\right) \tag{20}$$

Contrary to the AC model without proprioception, there is still a steady state compatible with the initial conditions. The apical angle of the steady state of the A_a model is given by

$$A(L, t \to \infty) = 0 \tag{21}$$

and the curvature of the steady state is simply defined by

$$C(t \to \infty) = A_0 L^{-1} \tag{22}$$

A_RC model

The local equation of purely local photogravitropism, called the local photo-gravi-proprio-ceptive equation is then given by

$$\frac{\partial C(s,t)}{\partial t} = -\nu\left(A(s,t) - A_P\right) - \beta A(s,t) - \gamma C(s,t) \tag{23}$$

A simple change of variables can be performed to simplify the equation 23

$$A'(s,t) = A(s,t) - A_P\frac{\nu}{\nu+\beta} = A(s,t) - A_R \qquad C'(s,t) = \frac{dA'(s,t)}{dt} = C(s,t) \tag{24}$$

where

$$A_R = A_P\frac{\nu}{\nu+\beta} \tag{25}$$

and with the dimensionless number M

$$A_R = A_P\frac{1}{1+M} \tag{26}$$

Equation 23 is then rewritten in a more compact form

$$\frac{\partial C(s,t)}{\partial t} = -(\nu+\beta)A'(s,t) - \gamma C(s,t) \tag{27}$$

with no constant parameter. Starting with the same set of initial conditions, equations 2, the initial condition to be considered are then

$$A'(s,0) = A'(s,t) = A'_0 = A_0 - A_R \qquad C'(0,t) = C(0,t) = 0 \tag{28}$$

This equivalent to the AC model where $\beta \to \beta+\nu$ and $A \to A - A_R$. The steady state is then defined by

$$A'(s,t) = A'_0 e^{-\beta s/\gamma} \tag{29}$$

$$A(s,t) = A_0 e^{-\beta s/\gamma} + A_R\left(1 - e^{-\beta s/\gamma}\right) \tag{30}$$

Indeed from equation 27 the apical angle of the steady state of the A_RC model is given by

$$A'(L,t\to\infty) = A'_0 e^{-B'} = A'_0 e^{-(B+D)} \tag{31}$$

$$A(L,t\to\infty) = (A_0 - A_R)e^{-(B+D)} + A_R \tag{32}$$

In the absence of light, $D = 0$, if the apical part of the organ reach the vertical, $A(L,t\to\infty) \sim 0$, it is expected that $e^{-B} \sim 0$. As the length of convergence is smaller when phototropism and gravitropism interacts than in the gravitropic case, $B + D > B$, the term $e^{-(B+D)}$ can therefore be neglected

$$A(L,t\to\infty) \sim A_R \tag{33}$$

Apical Photoception : $A_{Ra}C$ model

In the case where the perception of light is apical, the equation that describes this apical-photo/local-gravi-proprio-ception driven movement, called the $A_{Ra}C$ model, is given by

$$\frac{\partial C(s,t)}{\partial t} = -\nu\left(A(L,t) - A_P\right) - \beta A(s,t) - \gamma C(s,t) \tag{34}$$

with the same change of variable that was used previously (equation 24), the dynamical equation 36 can be simplified.

$$\frac{\partial C(s,t)}{\partial t} = -\nu A'(L,t) - \beta A'(s,t) - \gamma C(s,t) \tag{35}$$

The steady state is given by

$$\frac{\partial C(s,t)}{\partial t} = -\nu A'(L,t) - \beta A'(s,t) - \gamma C(s,t) = 0 \tag{36}$$

The general solution is given by

$$C(s,t) = K_1 e^{-\beta s/\gamma} \tag{37}$$

where K_1 is a constant, and the orientation is

$$A'(s,t) = K_2 e^{-\beta s/\gamma} \tag{38}$$

where K_2 is an other constant. The particular solution shuld solve

$$A'(s,t) = -\frac{\nu}{\beta} A'(L,t) \tag{39}$$

yielding the general solution of the equation.

$$A'(s,t) = K_2 e^{-\beta s/\gamma} - \frac{\nu}{\beta} A'(L,t) \tag{40}$$

The constant K_2 should be identified, as well as the apical angle. With the initial condition of a clamped organ (equation 2)

$$A'(0,t) = K_2 - \frac{\nu}{\beta} A'(L,t) \tag{41}$$

and then

$$K_2 = A'_0 - \frac{\nu}{\beta} A'(L,t) \tag{42}$$

The apical angle is then given by

$$A'(L,t) = \left(A'_0(0,t) - \frac{\nu}{\beta} A'(L,t) \right) e^{-B} - \frac{\nu}{\beta} A'(L) \tag{43}$$

yielding

$$A'(L,t) = A'_0 \frac{e^{-B}}{1 + M^{-1}\left(1 - e^{-B}\right)} \tag{44}$$

Finally the steady-state shape is described by the equation

$$A'(s, t \to \infty) = A'_0 \left(e^{-Bs/L} - e^{-B} \frac{1 - e^{-Bs/L}}{M + (1 - e^{-B})} \right) \tag{45}$$

$$C(s, t \to \infty) = -A'_0 \frac{B}{L} e^{-Bs/L} \left(1 + \frac{e^{-B}}{M + (1 - e^{-B})} \right) \tag{46}$$

1 Influence of a propagative term

The perception of the apical angle is not instantaneous along the stem but need to be propagated along the stem. This propagation is mediated by auxin. The speed of auxin propagation along the organ range from $0.5 mm.h^{-1}$ up to $20 mm.h^{-1}$ [1]. For example in the inflorescence stem of Arabidopsis, the speed of propagation ranges from $8 mm.h^{-1}$ to $15 mm.h^{-1}$.

The validity of the assumptions of an instantenous time of propagation time is tested with coleoptile where the perception of light is known to be apical []. The propagation speed in avena coloptile is

$c = 8 \pm 3mm.h^{-1}$ [1]. The time to propagate from the apex to the base is dependant on the length of the growth zone. Unfortunately this information is unavailable for oat coleoptile, but it has been shown that wheat coleoptile and oat coleoptile exhibit similar gravitropic behavior and similar size [2]. In wheat coleoptile, the length of the growth zone is $20 \pm 4mm$ [3]. This means that the time for auxin to go from the tip to the base is around $T_B = 2.5 \pm 0.3h$

The proprioceptive term tends to stabilise the movement and improve the convergence of the organ (Bastien2013) so it is expected that the photoceptive apical A_aC model without proprioception should be the most influenced by the propagation along the stem. This time should be compared with the characteristic time of the photoceptive process given by $Tc = (\dot{E}\nu L)^{-1}$ where $\dot{E}$ is the elongation rate of the organ [3]. There are very few to no measurements of the actuel elongation rate. However the elongation velocity c_E which represents the difference of length of the organ as a function of time ahas been measured. This velocity can be approximated by $\dot{E}L$ and has been found to be close to $0.5mm.h^{-1}$. In absence of shrinkage of the organ we expect $\nu \sim 1$ [3]. The ratio $T_B/T_C \sim 0.07$.

It is striking to see that as long as the propagation is faster than the characteristic time of the movement (Figure 1, 2, 3), the effects of the propagation on the movement is small. In the case of the avena coleoptile, the effect of the propagation are barely noticeable on the movement and on the final shape.

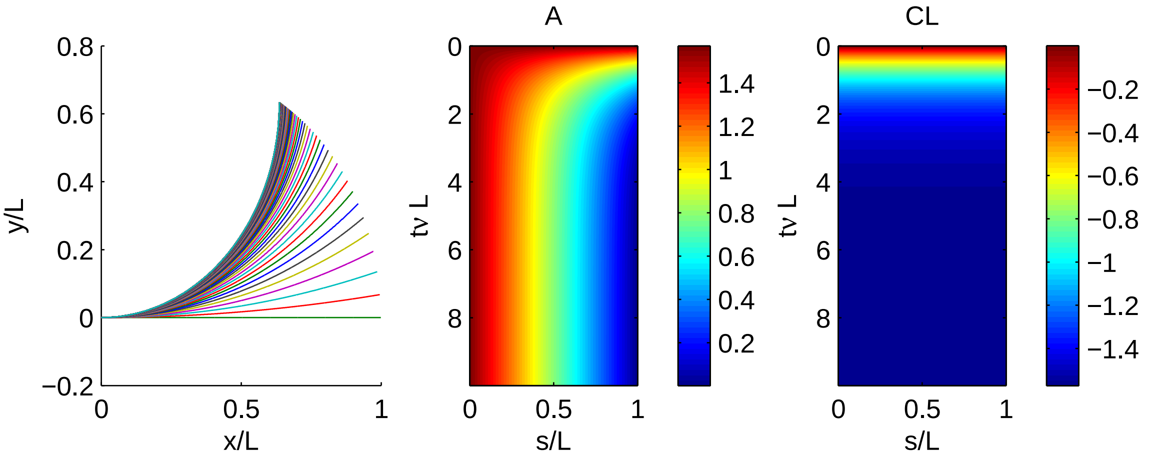

Figure 1. Solution of the A_aC model for instantaneous propagation, $T_B = 0$.

Supplementary References

References

1. Kramer EM, Rutschow HL, Mabie SS (2011) Auxv: a database of auxin transport velocities. Trends in plant science 16: 461–463.

2. Tarui Y, Iino M (1997) Gravitropism of oat and wheat coleoptiles: dependence on the stimulation angle and involvement of autotropic straightening. Plant and cell physiology 38: 1346–1353.

3. Bastien R, Douady S, Moulia B (2014) A unifying modeling of plant shoot gravitropism with an explicit account of the effects of growth. Frontiers in Plants Science 5.

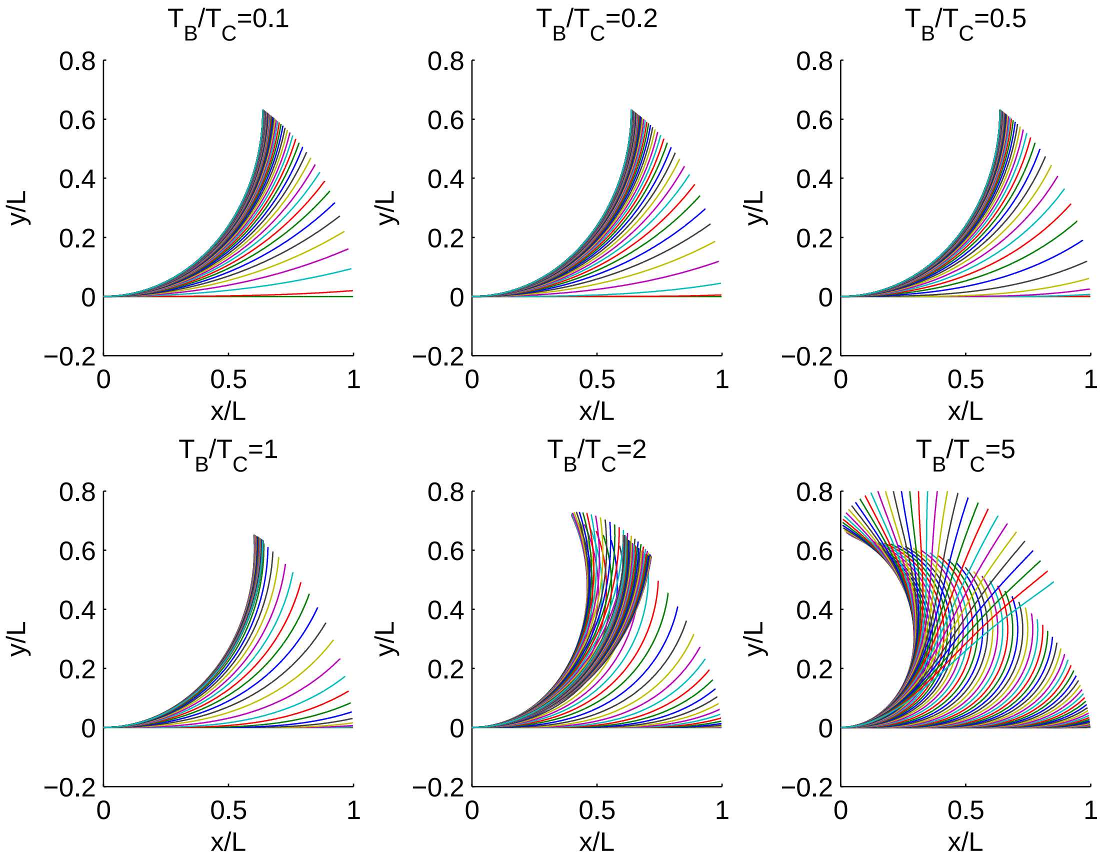

Figure 2. Simulation of the A_aC model for different value of the ratio T_B/T_C. As long as the the propagation is faster than the characteristic time of the movement, the solution is similar to the solution with instantaneous propagation.

4. Polyanin AD, Manzhirov AV (2012) Handbook of integral equations. CRC press.

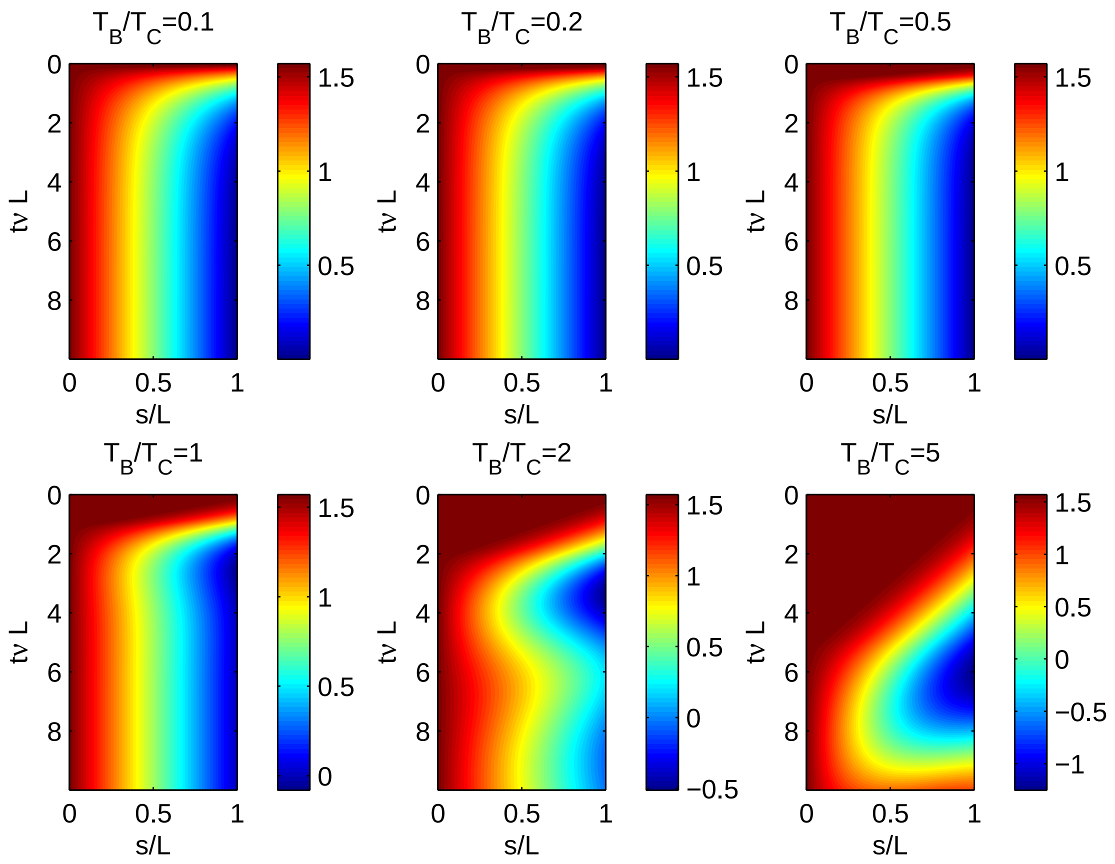

Figure 3. Orientation of the organ in the A_aC model for different value of the ratio T_B/T_C. As long as the the propagation is faster than the characteristic time of the movement, the solution is similar to the solution with instantaneous propagation.

Table 1. Limit cases of the $A_{Ra}C$ model

name	*acronyms*
$B \to \infty$	$A'(s) = 0$
$B \to 0$	$A'(s) = A'_0 \frac{1}{1+D}\frac{s}{L}$
$D \to \infty$	$A'(s) = A_0 \left(e^{-Bs/L} - \frac{1-e^{-Bs/L}}{e^B - 1} \right)$
$D \to 0$	$A'(s) = A'_0 e^{-Bs/L}$
$M \to \infty$	$A'(s) = A'_0 e^{-Bs/L}$
$M \to 0$	$A'(s) = A_0 \left(e^{-Bs/L} - \frac{1-e^{-Bs/L}}{e^B - 1} \right)$

Steady-state of the $A_R C$ model, in the different limit cases..
